# Tectonic Characteristics of the Juyanhai Depression in the Yingen-Ejinaqi Basin, Inner Mongolia, China, Based on Gravity, Electrical, and Seismic Data

**DOI:** 10.1021/acsomega.4c07755

**Published:** 2025-02-16

**Authors:** Haihong Xu, Junlin Zhou, Xiaofeng Han, Yuhong Li, Jianshe Wei, Bo Song, Jizhong Shi, Wei Xu, Chunguan Zhang, Baowen Wang, Fei Zhao

**Affiliations:** † Xi'an Center of Geological Survey, China Geological Survey, Xi'an 710119, China; ‡ Northwest China Center for Geoscience Innovation, Xi'an 710119, China; § School of Earth Sciences and Engineering, Xi'an Shiyou University, Xi'an 710065, China; ⊥ Key Laboratory of Paleozoic Oil and Gas Geology in Northern China, CGS, Xi'an 710119, China

## Abstract

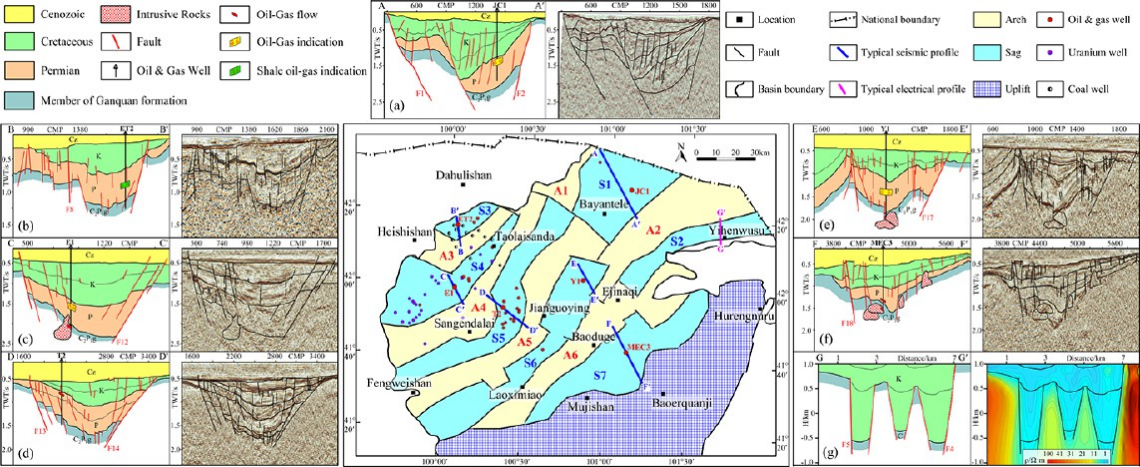

To determine the tectonic characteristics of the Juyanhai Depression in the west of the Yingen-Ejinaqi Basin, gravity data in the study area were processed and analyzed on the basis of the collection of existing geophysical data. Then, fault systems in the Juyanhai Depression were comprehensively presumed and explained mainly using processed gravity data in conjunction with the electrical and seismic data. Existing tectonic units in the depression were divided and studied afterward. There are mainly three groups of faults in the Juyanhai Depression, namely, the NE (or NNE)-, NW (or NWW)-, and nearly EW-trending ones. Faults not only control the thickness difference of sedimentary strata on both sides but also play an important role in controlling the formation of local structures and hydrocarbon accumulation. The Juyanhai Depression can be categorized into 13 primary structures, including six arches and seven sags. The sags and arches are independent, separated, and distributed in rows along the NE (or NNE) trend on the whole. According to structural patterns, sags can be classified into single-fault and double-fault ones. The characteristics of single-fault sags are that their formation is controlled by the main controlling normal faults of boundaries, manifested as half-graben fault depressions. The characteristics of double-fault sags are that their formation is controlled by the normal faults on both sides, while the fault distance of the sag-controlling faults differs on both sides. The research region has good prospects for a variety of resources, including oil and gas, shale oil and gas, coal, and uranium, and the distribution of these resources is closely related to the range of tectonic units. Therein, oil–gas exploration should mainly focus on sags. In the shallow part of the sags, fault-anticlines, fault blocks, fault-screened traps, and lithologic oil–gas reservoirs in the slopes should be the main targets of exploration. In the deep part of the sags, the bedrock buried hill reservoirs and the igneous rock lateral-screened oil–gas reservoirs formed by igneous intrusions are favorable exploration targets.

## 1 Introduction

**Figure 1:**
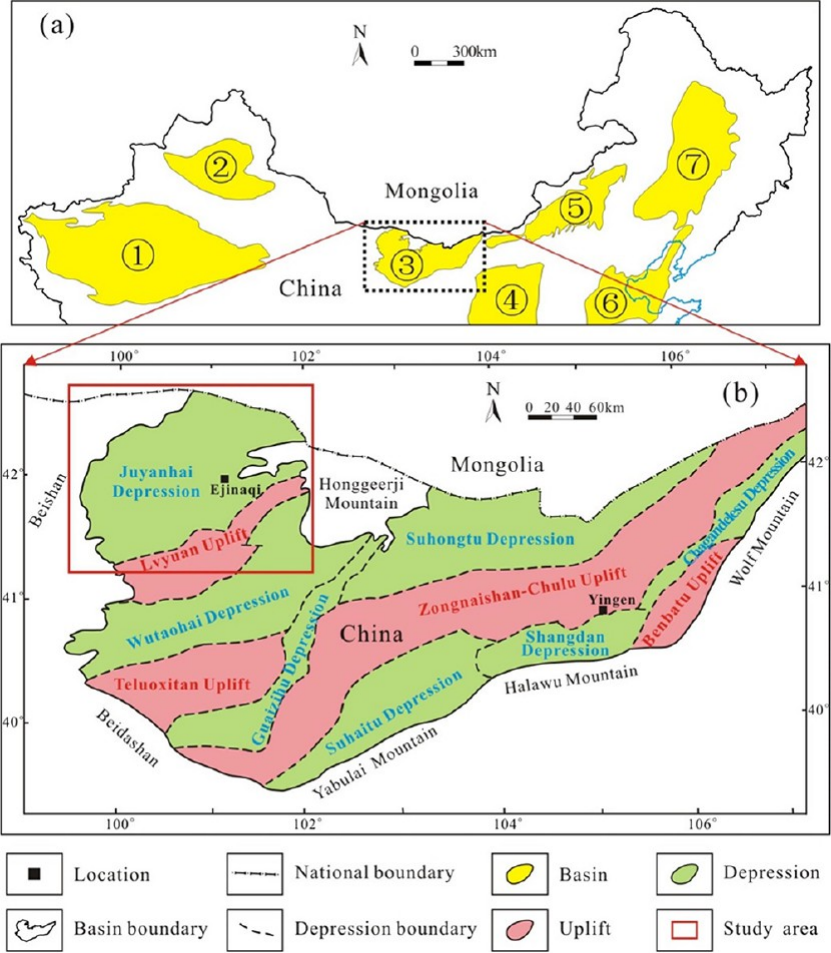
(a) Location map of the Yingen-Ejinaqi basin. Adapted with permission from ref (5). Copyright [Journal of Asian Earth Sciences, 2020]. ① Tarim Basin, ② Jungger Basin, ③ Yingen-Ejinaqi Basin, ④ Ordos Basin, ⑤ Erlian Basin, ⑥ Bohai Bay Basin, and ⑦ Songliao Basin. (b) Structural location map of the Juyanhai Depression. Adapted with permission from refs (2,3). Copyright [Petroleum Industry Press, 2006] and [Geological Publishing House, 2012].

The Yingen-Ejinaqi Basin, situated in the middle of Northern China, is a rare large- to medium-sized sedimentary basin subject to very little petroleum geological exploration for inland China (Figure 1a).^1−3^ Located in the northwest corner of Yingen-Ejinaqi Basin,^4,5^ the Juyanhai Depression is bounded by Lvyuan uplift in the southeast, borders with Honggeerji Mountain in the east, is adjacent to Beishan region in the west, and reaches the China–Mongolia border in the north (Figure 1b).

Since the 1990s, multiple organizations including North China Bureau of Petroleum Geology of the former Ministry of Geology and Mineral Resources, New Area Exploration Bureau of China National Petroleum Corporation, PetroChina Tuha Oilfield Company, Xi’an Center of Geological Survey of China Geological Survey, the Exploration and Development of Land and Resources of Inner Mongolia, and China National Nuclear Corporation have carried out geological surveys, geophysical investigation, and analysis and testing of rock samples^2,3^ on the Juyanhai Depression in terms of stratigraphic distribution,^6,7^ fault system,^8,9^ and tectonic evolution.^10,11^ The petroleum geological condition,^4,12−14^ geological conditions of shale oil and gas,^15−17^ occurrence conditions of coal-bearing strata,^18^ and sandstone-type uranium mineralization^19,20^ in the study area were investigated to varying degrees. The research results show a good exploration prospect for various resources in the Juyanhai Depression.

Previous researchers have obtained industrial oil–gas flows in sags such as Tiancao, Lujing, and Jigeda.^2−4,21^ Coal seams and good shale oil and gas displays have been discovered in Wuzhuer sag and its adjacent areas.^16,18^ Uranium mineralization clues have been discovered in the southern part of Lujing sag.^19,20^ According to their planar location, various resources such as oil and gas, shale oil and gas, coal, and uranium discovered in the Juyanhai Depression are closely related to the distribution of tectonic units in the study area. However, due to the low exploration level of the Juyanhai Depression, the boundaries and planar distribution ranges of tectonic units divided by different scholars vary greatly,^2−4^ which affects the investigation and evaluation of various resource explorations. In addition, due to the significant differences in exploration levels among different sags, existing research has mostly focused on individual sags with high levels of well and seismic exploration, such as Tiancao sag and Lujing sag,^22−24^ with little overall evaluation of the structural units in the study area. There is a lack of systematic research on the fault system and structural characteristics of the study area, which limits an accurate understanding of the geological features of the Juyanhai Depression.

In view of this, the gravity data and newly acquired electrical and seismic data of the research region were collected. Gravity anomalies were characterized by processing the gravity data. Based on gravity anomalies, geological structures in the Juyanhai Depression were investigated in conjunction with abnormal changes in geologic bodies, as described by electrical and seismic profiling data. The fault systems in the research region were presumed, the division scheme of tectonic units was determined and refined, and the tectonic framework and development characteristics in the region were revealed. Moreover, vertical and horizontal geological structures and their developmental characteristics in each sag were studied. The research provides abundant geophysical data and evidence for the exploration of multiple resources in the region.

## 2 Geological and Geophysical Characteristics

### 2.1 Stratigraphic Distribution and Development Characteristics

**Figure 2:**
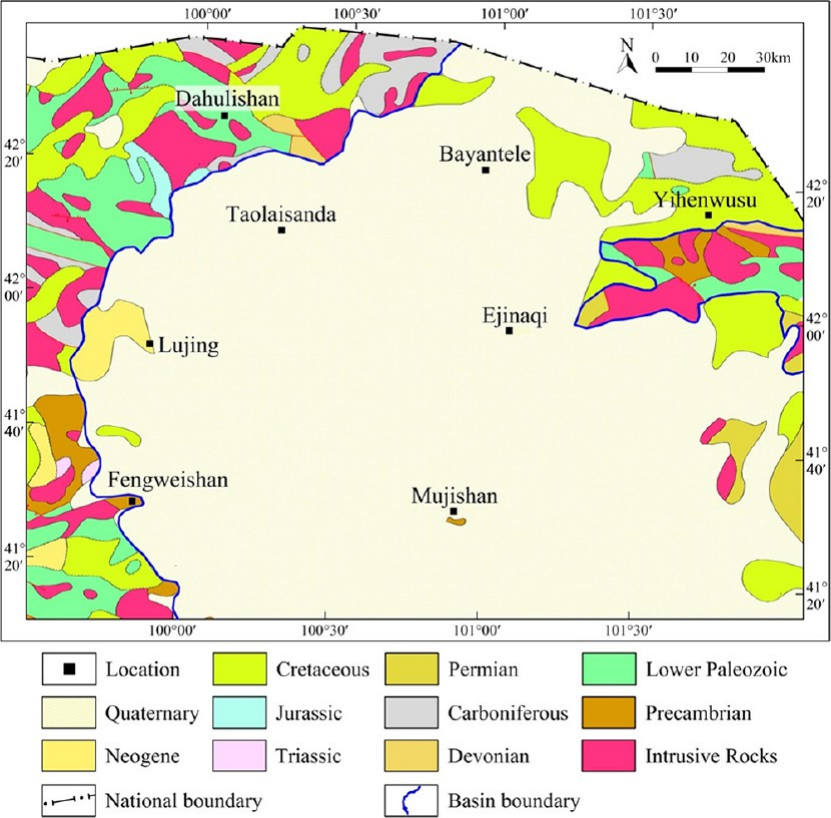
Geological map of the Juyanhai Depression and adjacent areas.

Archaeozoic, Proterozoic, Palaeozoic, Mesozoic, and Cenozoic strata are well developed in the Juyanhai Depression and its adjacent regions (Figure 2). Archaeozoic and Palaeoproterozoic strata constitute the ancient fold basement,^25^ and the sedimentary cover includes Palaeozoic, Mesozoic, and Cenozoic strata. Palaeozoic sedimentary strata mainly comprise Carboniferous-Permian system; Mesozoic sedimentary strata are composed of Triassic, Jurassic, and Cretaceous systems from the bottom up; and Cenozoic sedimentary strata are Paleogene-Neogene and Quaternary systems.^8^ Therein, the Carboniferous-Permian system is widely distributed; the Triassic system is only developed in local areas; the Jurassic system is distributed across a wider range than the Triassic system while remaining limited; the Cretaceous system shows a wide distribution throughout the depression; the Paleogene-Neogene system is not fully developed and is exposed at the basin margin; and the Quaternary system is extensively distributed while being relatively thin.^2^

**Figure 3:**
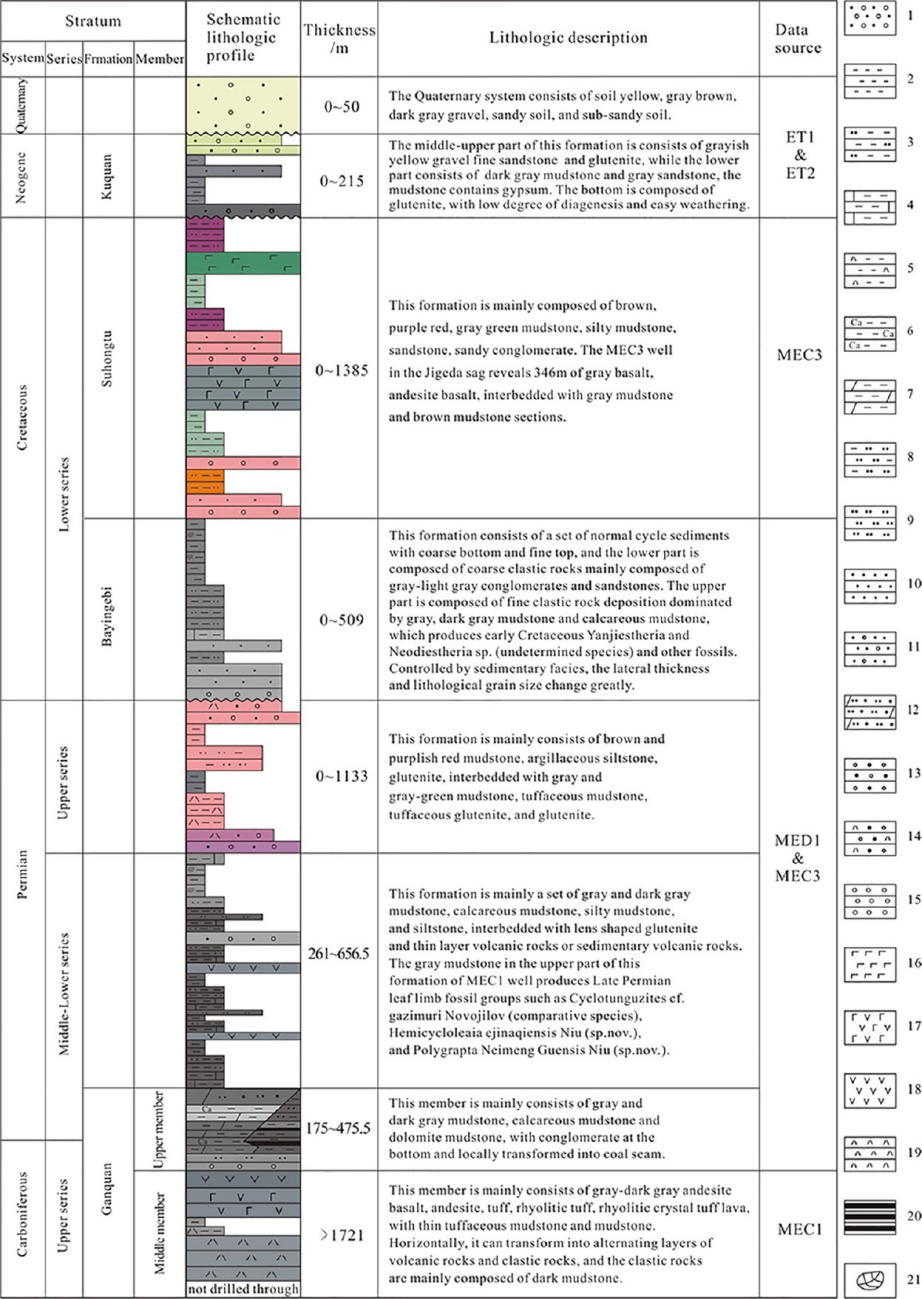
Comprehensive stratigraphic column of the wells of the Juyanhai Depression. Adapted with permission from ref (21). Copyright [Geology in China, 2023]. 1-Sandy gravel, 2-Mudstone, 3-Silty mudstone, 4-Lime mudstone, 5-Tuffaceous mudstone, 6-Calcareous mudstone, 7-Dolomitic mudstone, 8-Argillaceous siltstone, 9-Siltstone, 10-Sandstone, 11-Gravel-bearing sandstone, 12-Dolomitic siltstone and fine sand, 13-Sandy conglomerate, 14-Tuffaceous sandy conglomerate, 15-Conglomerate, 16-Basalt, 17-Andesite basalt, 18-Andesite, 19-Tuff, 20-Coal seam, and 21-Conchostracan.

Comparison results of stratigraphic division of wells are shown in Figure 3.^21^ The following strata are developed from top to bottom of the Juyanhai Depression: the Quaternary system (Q_3+4_), Neogene Kuquan formation (N_2_k), lower Cretaceous Suhongtu formation (K_1_s), lower Cretaceous Bayingebi formation (K_1_b), upper Permian series (P_3_, ungrouped), middle-lower Permian series (P_1–2_, ungrouped), upper member of Ganquan formation in the upper Carboniferous-lower Permian series (C_2_P_1_g^3^), and middle member of Ganquan formation in the upper Carboniferous-lower Permian series (C_2_P_1_g^2^). The Quaternary system has a thickness of 0–50 m and shows unconformable contact with underlying strata; the Neogene Kuquan formation is 0–215 m thick and demonstrates an unconformable contact with underlying strata. The thickness of the Cretaceous system is 0–1894 m, among which the thickness of the lower Cretaceous Suhongtu formation is 0–1385 m, and the thickness of the lower Cretaceous Bayingebi formation is 0–509 m. The Cretaceous system is the main hydrocarbon-bearing layer in the region and also a favorable layer for uranium enrichment and mineralization.^15,19,20^ It has an unconformable contact with underlying strata; upper Permian series with a thickness of 0–1213 m shows conformable or parallel unconformable contact with underlying strata; 261–656.5 m thick middle-lower Permian series, exhibiting favorable indication of shale oil and gas,^16^ is also the main hydrocarbon-bearing layer in the region and has a conformable contact with underlying strata; with a thickness of 175–475.5 m, the upper member of Ganquan formation in upper Carboniferous-lower Permian series is an important hydrocarbon-bearing layer and also an important coal-bearing section in the region,^18^ showing a conformable contact with underlying strata; and the middle member of Ganquan formation in upper Carboniferous-lower Permian series has a thickness exceeding 1721 m (not drilled through) and is a notable hydrocarbon-bearing layer.^21^

### 2.2 Formation Density and Electrical Characteristics

To understand physical characteristics of the research region and provide a basis for the qualitative and quantitative interpretation of data, existing physical research data were collected to further analyze changes in the density and resistivity of different formations (rocks).

The comprehensive densities of the Cenozoic and Mesozoic strata are 2.19 × 10^3^ and 2.51 × 10^3^ kg/m^3^, respectively, and that of the Upper Palaeozoic Carboniferous-Permian systems is 2.66 × 10^3^ kg/m^3^. In addition, the comprehensive density of the Devonian and Lower Palaeozoic systems is 2.73 × 10^3^ kg/m^3^ and that of the Proterozoic strata is 2.75 × 10^3^ kg/m^3^.^26^

The density and thickness of each stratigraphic unit were adopted to assess their influences on the interpretation of the gravity data. Cenozoic strata are regarded as a typical low-density layer that shows a significant difference in density from the Mesozoic strata. Whereas due to the thin nature of the Cenozoic strata and slight undulation of its bottom interface, the resulting gravity anomaly has a small amplitude and is gentle, the influence of which can be ignored in most areas. The Mesozoic strata and Upper Palaeozoic Carboniferous-Permian systems show a density difference of 0.15 × 10^3^ kg/m^3^ and provide the most important density interface in the region of interest. It can serve as an important basis for the use of gravity data to explain the thickness, fluctuation of the bottom interface, and burial depth of Mesozoic strata. The Upper Palaeozoic Carboniferous-Permian systems have a density difference of 0.07 × 10^3^ kg/m^3^ with Devonian-Lower Palaeozoic formation, representing another important density interface in the region. It can be used as an important basis of gravity data for explaining the thickness, undulation of the bottom interface, and burial depth of the Carboniferous-Permian system.

An electrical model of strata is established based on the first branch of magnetotelluric sounding (MT) curves and the stratigraphic unit to which it belongs.^27^ Apparent resistivity profiles obtained from MT reflecting vertical resistivity changes of strata with depth are the basic data pertaining to electrical stratification. The resistivities of different stratigraphic units in the area and adjacent regions were obtained through the arrangement and statistics of existing data.

**Figure 4:**
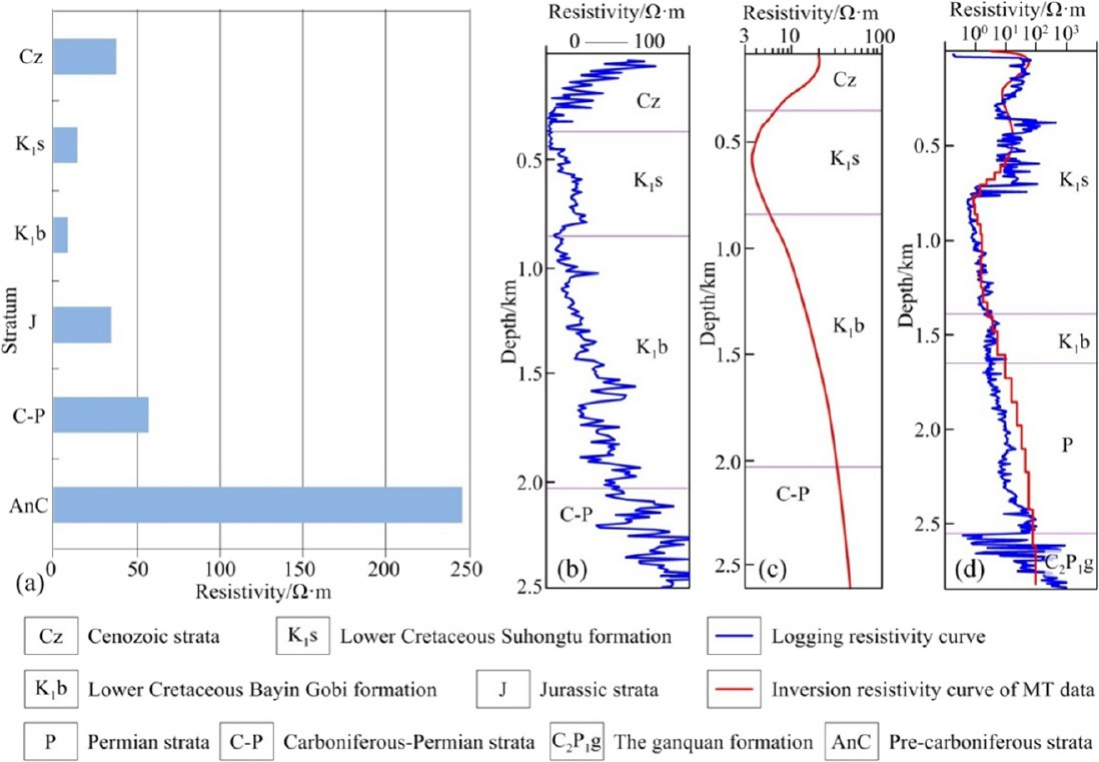
Electrical characteristics of the research area. Adapted with permission from refs (3,27,28). Copyright [Geological Publishing House, 2012], [Geological Bulletin of China, 2011], and [Geophysical Prospecting for Petroleum, 2022]. (a) Resistivity of the main stratum in the surface outcrop area; (b) logging resistivity curve of well T2; (c) inversion resistivity curve of MT data near well T2; and (d) logging resistivity curve and MT inversion resistivity curve of well MEC3.

Analysis reveals that Cenozoic strata are widely exposed, while their thickness varies remarkably, characterized by a high resistivity, which is generally 10–80 Ω·m and 38 Ω·m on average; the resistivity of the Cretaceous Suhongtu formation ranges from 5 to 30 Ω·m and its average is 14 Ω·m, while that of the Cretaceous Bayingebi formation is in the range of 2–25 Ω·m and 9 Ω·m on average, with the Cretaceous system featured by a low resistivity on the whole; the Jurassic system has a resistivity of 20–50 Ω·m, which is 35 Ω·m on average, and is manifest as a subhigh-resistivity layer; the resistivity of the upper Palaeozoic Carboniferous-Permian system is in the range of 30–70 Ω·m and 58 Ω·m on average, which is slightly higher than that of the Jurassic system; the Pre-carboniferous system shows a resistivity of 100–600 Ω·m, which is 245 Ω·m on average, and is characterized by a high resistivity; intrusive rocks and lava differ greatly in the resistivity, which changes in the range of 80–2000 Ω·m and is 1250 Ω·m on average, and they are generally characterized by ultrahigh resistivity (Figure 4a).^3,26,27^

The resistivity–depth curves were obtained in inversion results of near-well MT data from existing wells in the research region and then compared with the resistivity logging curves. The results show that the two are consistent in terms of shape, which reflects changes in the resistivity of different stratigraphic units (Figure 4b–d).^27,28^ Combining the first branch of MT curves, near-well MT data, and resistivity logging data, electrical characteristics of various main stratigraphic units in the regions were revealed: the Cenozoic system is a set of strata with a relatively high resistivity; the Cretaceous system is a low-resistivity marker layer and the Jurassic system is a subhigh-resistivity layer; the upper Palaeozoic strata form a subhigh-resistivity layer; and the lower Palaeozoic and strata there under are a high-resistivity layer.^3^

### 2.3 Characteristics of Seismic Responses

**Figure 5:**
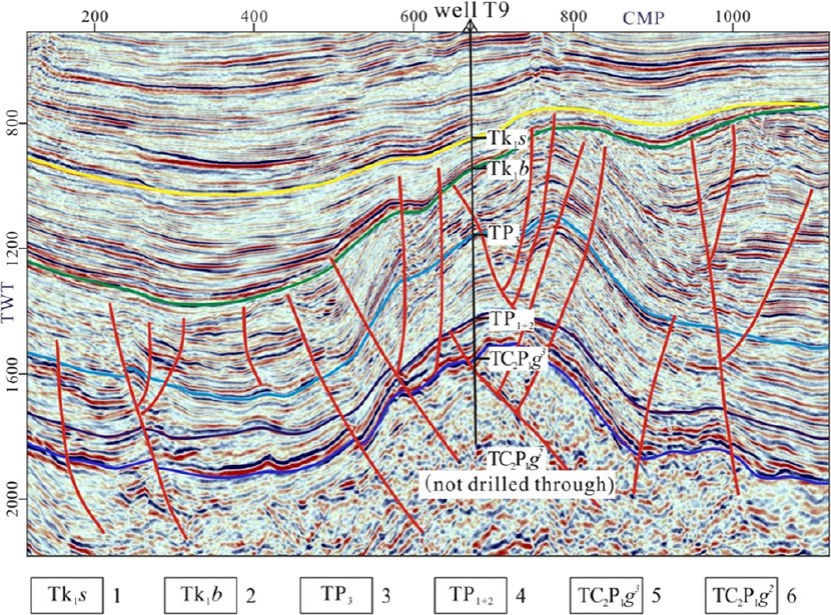
Reflection characteristics of the seismic profile through well T9. Adapted with permission from ref (21). Copyright [Geology in China, 2023]. 1- Bottom interface of Suhongtu Formation, 2- bottom interface of Bayingebi Formation, 3- bottom interface of Upper Permian, 4- bottom interface of Medium-Lower Permian, 5- bottom interface of Upper Ganquan Formation, and 6- bottom interface of Middle Ganquan Formation.

After sedimentation of the Carboniferous-Permian system, the Juyanhai Depression had experienced long-term uplift and denudation as well as multistage tectonic reworking, until receiving sediments in the Cretaceous period.^21^ The Cretaceous system is widely distributed in the sags. The bottom interface of the cretaceous shows angular unconformable contact with underlying strata, which is a regional unconformity surface that can be compared and traced in the whole region.^7^ According to seismic profiles (Figure 5), the seismic reflection sequences above and below the unconformity differ greatly: Reflection sequences below the unconformity have medium-high amplitudes and short-axis seismic events and are obviously cut by the upper structural layer, so their continuity is poorer than that of the upper structural layer; those above the unconformity have medium and high amplitudes and favorable continuity, with reflection sequences appearing alternately. The upper and lower structural layers differ significantly in the characteristics of wave impedance. In the lower structural layer, the strata have a large dip angle and change greatly transversely, while those are gentle in the upper structural layer and stable in the transverse direction.

Combining with stratigraphic lithologic association at well T9, seismic reflection characteristics of main stratigraphic units in the two structural layers were analyzed from the bottom up (Figure 5).^7,21^ (1) For the lower structural layer: ① The middle member of the Ganquan formation (C_2_P_1_g^2^) is 433.0 m thick (not drilled through) and mainly contains volcanic rocks and sedimentary volcanic rocks interbedded with clastic rocks. The seismic reflection energy is unbalanced and intermittent, and seismic reflection information is so poor that it does not have an obviously continuous reflection axis, which align with characteristics of massive volcanic rocks. ② The upper member of the Ganquan formation (C_2_P_1_g^3^) has a thickness of 131.0 m and mainly comprises conglomerate rocks, interbedded with mudstone and silty mudstone. Seismic data indicate strong, peristaltic seismic reflection sequences. ③ The middle-lower Permian series (P_1+2_) is 1276.0 m thick and mainly includes mudstone, calcareous mudstone, silty mudstone, and argillaceous siltstone, interbedded with glutenite. Seismic reflection indicates weak reflection sequences with good continuity. ④ The upper Permian series (P_3_) has a thickness of 241.0 m. Its lower part mainly contains purple–red and brown mudstone interbedded with argillaceous siltstone; the upper part is mainly composed of glutenite, interbedded with purple–red and reddish–brown mudstone and silty mudstone. Seismic reflection sequences are relatively strong and peristaltic, with moderate to good continuity. (2) For the upper structural layer: ① the Bayingebi formation (K_1_b) has a thickness of 93.0 m, with gray glutenite in the lower part, and the upper part is interbedded of gray mudstone, silty mudstone, and argillaceous siltstone. Seismic reflection sequences are strong in the lower part, where subparallel–parallel reflection sequences with low-medium frequency and medium-high amplitude are developed; those in the upper part are relatively weak, with good continuity. ② The Suhongtu formation (K_1_s) mainly contains brown–brownish red mudstone and silty mudstone interbedded with argillaceous siltstone, intercalated with gray mudstone, silty mudstone, and argillaceous siltstone. Seismic reflection sequences are strong and show good continuity.^21^

## 3 Data Overview and Processing Methods

### 3.1 Data Sources and Distribution

The 1:200,000 gravity data cover most of the region of interest, except for local areas near the national border. The collected gravity data were digitized and interpolated into regular grid data (1 km × 1 km) using the radial basis function method. To reduce the influence of the boundary effect during data processing, the original data were extended via iterative interpolation,^29^ finally forming gridded data in the range of about 3.55 × 10^4^ km^2^.

**Figure 6:**
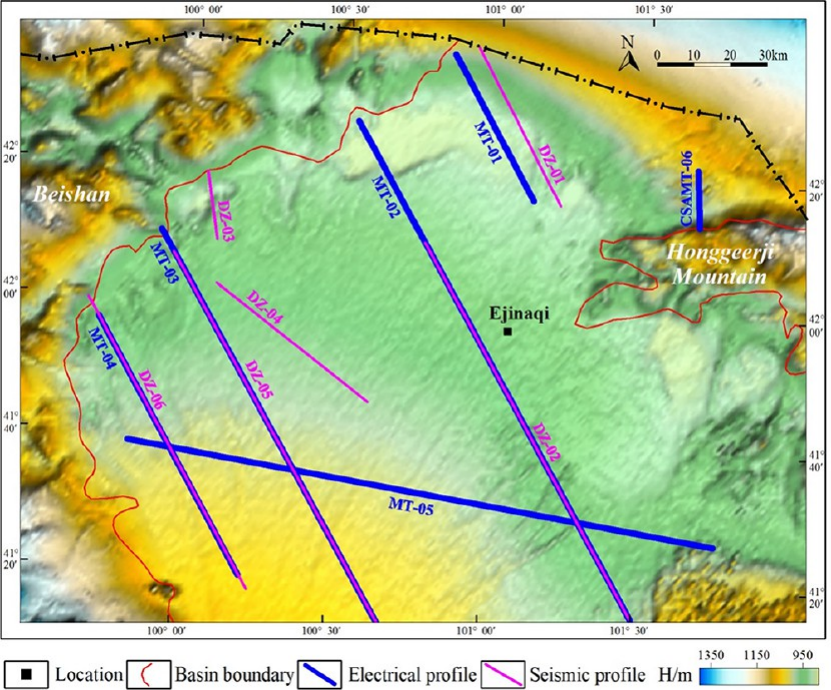
Location map of electrical and seismic profiles in the Juyanhai Depression.

Since 2007, China Geological Survey has conducted petroleum geological survey and regional geological and mineral survey in Ejinaqi and adjacent regions and implemented a large number of electrical and seismic surveys.^3,27,30−33^ Based on data from a petroleum geological survey of the Yingen-Ejinaqi Basin and aiming to reveal the geological structures of the Juyanhai Depression from an overall perspective, six electrical profiles and six seismic profiles were collected in the research region (Figure 6). Among them, five electrical profiles (MT-01, MT-02, MT-03, MT-04, and MT-05) were collected in the petroleum geological survey of the Yingen-Ejinaqi Basin. V5–2000 MT instruments were used to acquire field data through array measurement (CEMP), and the instruments were arranged in a cross shape with a point spacing of 500 m and observation frequency in the range of 320.000–0.001 Hz.^27^ CSAMT6 is a newly collected electrical profile in the regional geological and mineral survey, along which a GDP-32 multifunctional electrical prospecting apparatus was adopted for in situ data acquisition. Equator dipole devices were arranged, with the point spacing of 40 m and observation frequency in the range of 8192–1 Hz.^30^ Additionally, one seismic profile (DZ-01) was collected previously; the other five (DZ-02, DZ-03, DZ-04, DZ-05, and DZ-06) were collected by the petroleum geological survey project of the Yingen-Ejinaqi Basin, and vibroseismic excitation was mainly applied in the field. The total lengths of electrical and seismic profiles were 580 and 480 km, respectively. Therein, the locations of three seismic profiles overlapped the three electrical profiles.

### 3.2 Processing of Gravity Data

Gravity anomalies embody the Earth’s natural field and include overlapped information on gravitational effects of all underground geologic bodies. To characterize deep and shallow gravity anomalies and the structural variation associated therewith, highlighted details of local geologic bodies, gradient belts, and anomaly changes, gravity anomalies were subjected to separation, derivation, and filtering. Upward continuation was used to separate anomalies,^34,35^ thus attaining residual gravity anomalies,^36^ and vertical second derivative (VSD) was used to highlight boundaries of local geologic bodies^37^; second-order directional derivative along the horizontal direction and total horizontal derivative (THD) were used to highlight linear anomalies and locations of their horizontal boundaries.^38,39^ The further to underline zonal and linear variation characteristics of gravity anomalies, small subdomain filtering was used for structure-enhancing filtering of gravity anomalies to enhance boundary characteristics of anomalies in different zones.^40,41^

**Figure 7:**
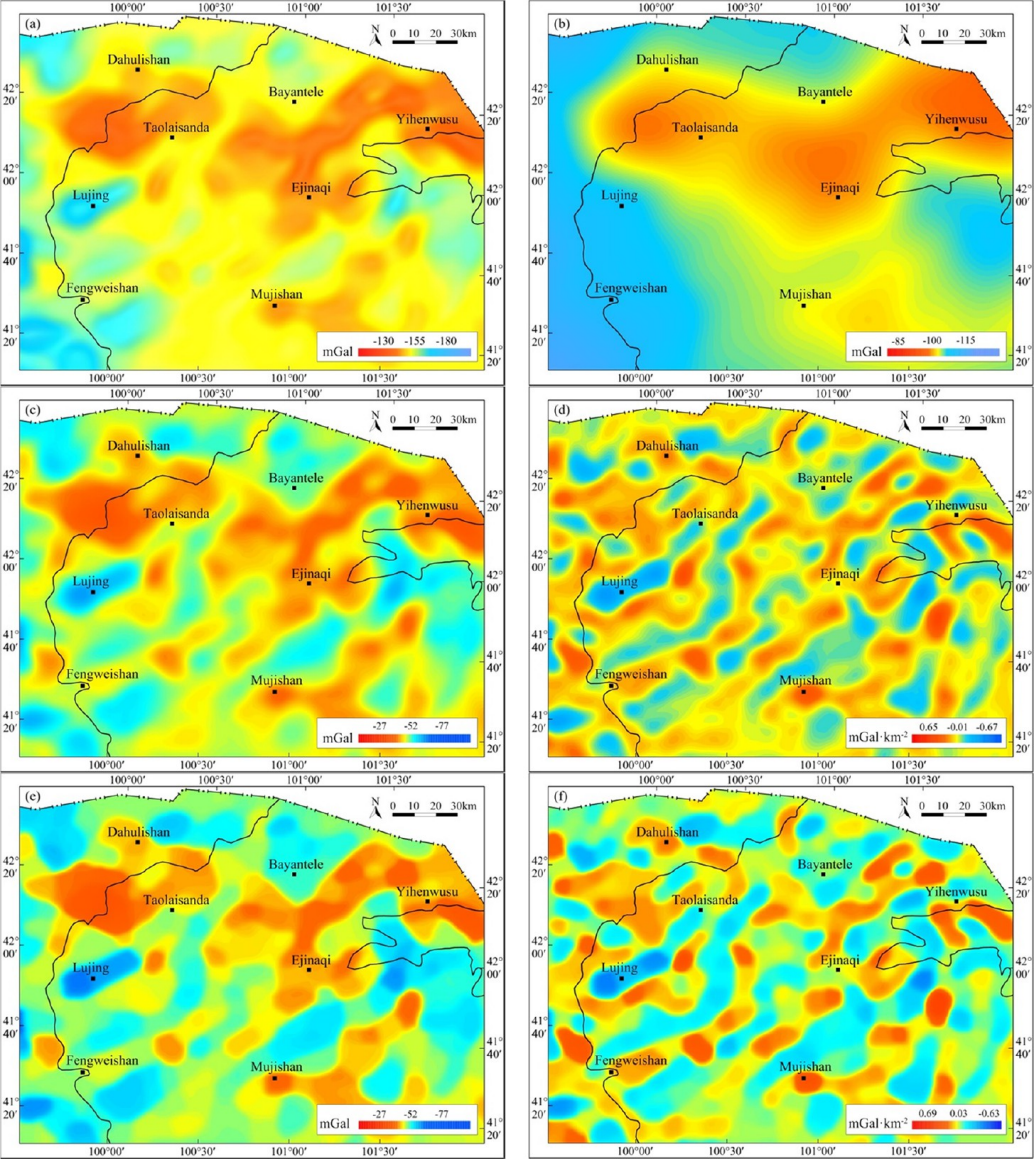
Gravity anomalies and their separation anomalies in the Juyanhai Depression and adjacent areas. (a) Bouguer gravity anomaly, (b) regional gravity anomaly by upward continuation for 15 km, (c) residual gravity anomaly, (d) vertical second derivative anomaly, (e) residual gravity anomaly with small subdomain filtering, and (f) vertical second derivative anomaly with small subdomain filtering.

Previous research has shown that during upward continuation, gravity anomalies of geologic bodies with a shallow burial depth and a small scale attenuate faster than those with a deep burial depth and a large scale.^34,35,42^ Therefore, upward continuation is also characterized by low-pass filtering and is generally applied to filter out the shallow part and thus highlight gravity anomalies of deep geologic bodies. Therefore, the upward continuation method is often used for the separation of anomalies. Whether a more reasonable regional anomaly can be distinguished by using the upward continuation method depends on the selection of the upward continuation height. To select an appropriate upward continuation height, multiple different heights (such as 5, 10, 15, 20, and 25 km) of gravity anomalies were processed. According to the research of Xu et al.,^36^ the anomaly with an upward continuation of 15 km was selected as the regional gravity anomaly in the study area. Upward continuation results reflect gravity anomalies of deep geologic bodies (Figure 7b). The residual anomaly (Figure 7c) was obtained by subtracting the upward continuation anomaly from the Bouguer gravity anomaly (Figure 7a). Compared with the Bouguer gravity anomaly, the residual gravity anomaly can reveal gravity anomalies of shallow geologic bodies, which further reveal the distribution of local geological structures after solving the VSD (Figure 7d). After structure-enhancing filtering of the residual anomaly and anomaly obtained by VSD, the shape and development of local structures were further highlighted. Figure 7e,f shows that the residual gravity anomaly in the research region shows an overall NE (NNE) trend and high and low gravity anomaly zones are distributed alternately in rows.

**Figure 8:**
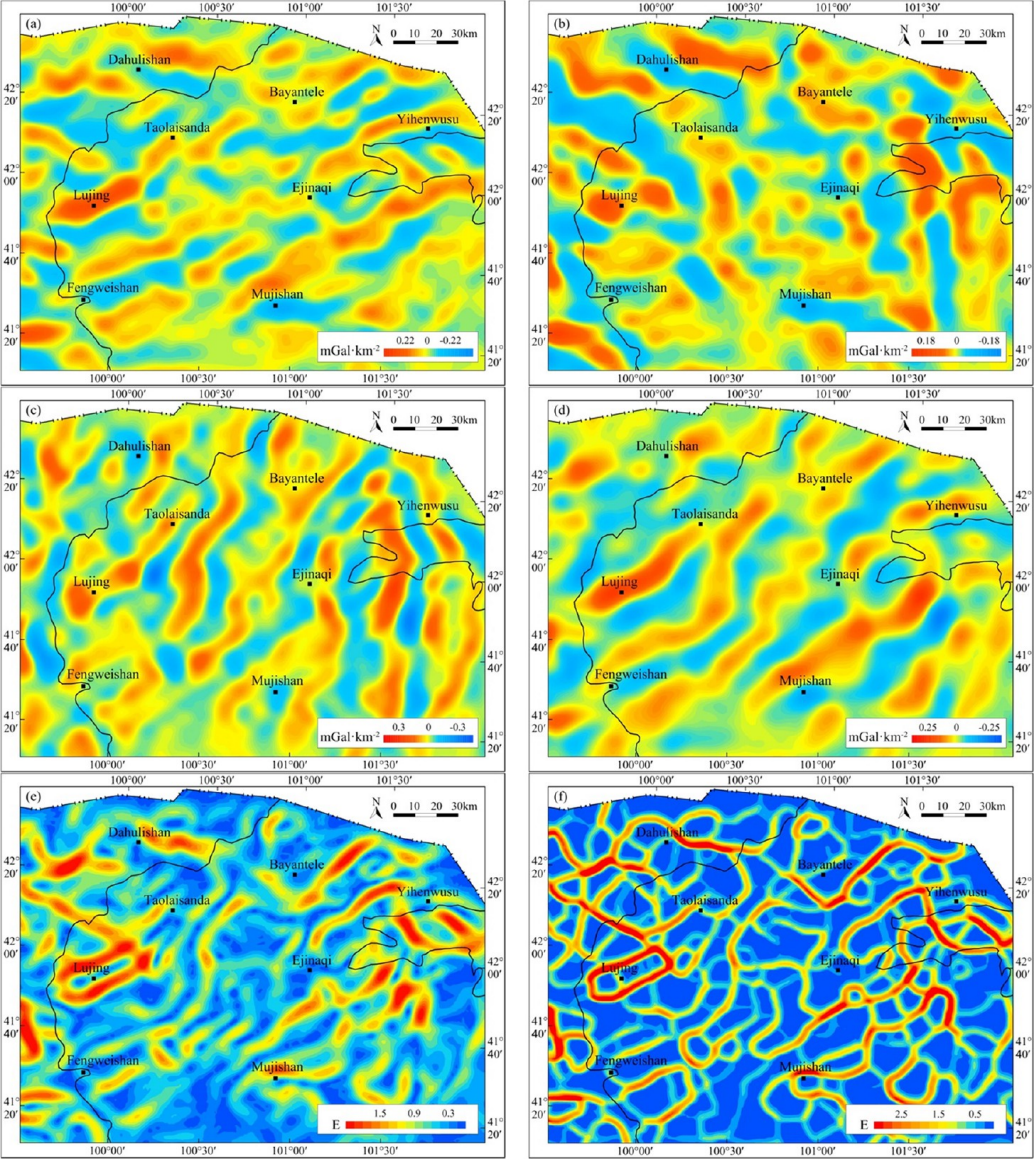
Gravity linear anomaly map of the Juyanhai Depression and adjacent areas. (a) Horizontal second direction (0°) derivative anomaly, (b) horizontal second direction (45°) derivative anomaly, (c) horizontal second direction (90°) derivative anomaly, (d) horizontal second direction (135°) derivative anomaly, (e) total horizontal derivative anomaly, and (f) total horizontal derivative anomaly after small subdomain filtering.

The horizontal derivative is mainly calculated to identify the fault strike, determine fault systems, and delimit the boundaries of local anomalies by cooperation with the vertical derivative. As illustrated in Figure 8a–d, horizontal derivatives in different directions reveal development characteristics of linear anomalies in different directions. The location of the horizontal boundary of faults can be deduced based on the zero curve of the second derivative. Moreover, the THD of gravity anomalies is maximized on the vertical physical boundary,^43^ so it can be used to highlight vertical boundaries of different lithological distribution areas (Figure 8e,f). Therefore, the use of processing results of the THD can better identify faults and other linear structures from gravity anomalies. The results of first structure-enhancing filtering and then calculating THD can better reveal salient characteristics of anomalies of linear structures in the region.^36,41^

### 3.3 Characteristics of Electrical and Seismic Profiles

**Figure 9:**
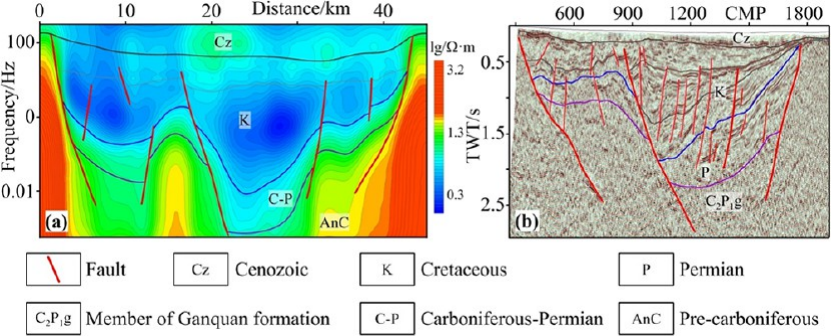
(a) MT-01 electrical profile and (b) DZ-01 seismic profile. DZ-01 adapted with permission from ref (10). Copyright [School of Geoscience Yangtze University, 2013].

Electrical and seismic profiles are key to understanding vertical development characteristics of geological structures in the research region. Based on previous methods and results,^3,8,10,27,30,36,40^ the present research entailed a comprehensive study of the collected electrical and seismic data. Among them, the electrical profile is mainly obtained according to the following steps: ① Static displacement correction and terrain corrections are applied using three methods of curve translation, regularization filtering, and spatial filtering to eliminate the distortion of resistivity curves caused by static displacement and the influence of the terrain. ② The residual static effect correction is performed using phase converted resistivity data. ③ A one-dimensional Bostick model is established under the constraints of rock and formation electrical, drilling, and geological data to perform two-dimensional inversion of continuous media with terrain, and the inversion results are forward fitted. ④ Repeating human–computer interaction forward and inversion under various constraints can be applied until objective and reasonable inversion results are obtained.^27^ The seismic profile is mainly acquired through the following steps: ① The static correction problem of seismic data is solved using the datum static correction technology and residual static correction technology of reflected waves. ② Using prestack multidomain frequency division and partition denoising technology to remove various types of noise and improve the signal-to-noise ratio of the data. ③ Surface consistent amplitude compensation technology is used to improve the imaging quality of seismic data. Through fine velocity analysis and multiple iterations, the continuity and imaging accuracy of the profile are improved. ④ The final profile is obtained by using prestack imaging to constrain the poststack migration imaging velocity field. In order to better use electrical and seismic data for data interpretation and identify characteristics of geological structures, two profiles at the same sag were used to compare the electrical and seismic data (Figure 9).

Figure 9a shows that resistivity on MT-01 electrical profile changes in a relatively high, low, subhigh, and high trend on the whole from shallow to deep part. ① The low-resistivity zone corresponds to the Mesozoic Cretaceous system, representing the most obvious low-resistivity marker layer and reflecting electrical characteristics of Mesozoic strata. According to this, the bottom interface of the Cretaceous system can be delineated, and the development degree of the low-resistivity zone reflects the sedimentation thickness of the Cretaceous system. ② The subhigh-resistivity zone is the Carboniferous-Permian system. The bottom interface of the Carboniferous system is determined in accordance with the high-resistivity marker layer of the Pre-carboniferous system, and the vertical extension of the subhigh-resistivity zone reflects the thickness of the Carboniferous-Permian system. ③ The high-resistivity zones correspond to arches (or uplifts). The absence of the low-resistivity marker layer in the medium-high-frequency band on the apparent resistivity curve and the rapid rise in the abnormal value on the curve indicate that the low-resistivity Mesozoic strata are absent or thin. Basement uplift causes the rapid ascent of apparent resistivity in the high frequency band. The high-resistivity basement of the Pre-carboniferous system undulates, and high-resistivity blocks are broken in local areas.

Figure 9b shows that the DZ-01 seismic profile clearly unveils the regional unconformity between the lower Cretaceous series and Permian system. Subparallel–parallel reflection sequences with medium-low frequency and medium-high amplitude are developed at the bottom of the Bayingebi formation, which exhibit good continuity in most areas of the research region while being chaotic in local areas. The bottom interface shows angular unconformable contact with underlying strata. Permian system shows a set of parallel or subparallel reflection sequences with alternated distribution of strong and weak ones, which reflects stratigraphic sequence dominated by sandstone and mudstone. The seismic reflection energy of the underlying Carboniferous system is unbalanced and intermittent, which shows reflection characteristics of strata with diverse lithologies including megaclasts, mudstone, volcanic breccia, tuffaceous mudstone, and andesitic tuff.

Comparison of the DZ-01 seismic profile and MT-01 electrical profile reveals that multiple faults are developed at the location passed by the profile, and both sides of faults differ greatly in terms of characteristics of resistivity and seismic reflection. These faults cut the basement into multiple blocks, thus forming a tectonic pattern with an alternated distribution of sags and arches. In sags, the electrical profile is generally characterized by a low resistivity, while the signal-to-noise ratio (SNR) of the seismic profile is high, where reflection sequences are well-developed in the shallow, middle, and deep parts. In arches, the electrical profile mainly shows characteristics of subhigh and high resistivity on the whole, while the SNR is low for the seismic profile, with clear seismic reflection in the shallow part, while there is an absence of obvious seismic reflection or chaotic seismic reflection in the middle and deep parts. According to the analysis, the multiple faults developed in the region are considered to control the boundaries of sags and arches as well as the sedimentation and distribution of Mesozoic strata and the Carboniferous-Permian system.

## 4 Characteristics of Fault Systems

### 4.1 Basis of Fault Identification

Faults are one of the most important tectonic activities in the Yingen-Ejinaqi Basin. They control the formation and distribution of arches and sags and also play a crucial role in sedimentation and oil–gas migration and accumulation.^2^ The occurrence of faults generally leads to nonuniform spatial distribution of the formation density, electrical property, and elasticity. However, the nonuniform changes in the formation density, electrical property, and elasticity cause variations of gravity anomalies, resistivity anomalies, and reflection events on seismic profiles.

Signs indicating the presence of faults in the gravity anomaly map include the dense gravity gradient belts, transition zone between linear gravity highs and lows, an obviously dislocated part of the axis of gravity anomalies, locations of both sides or axis of beaded anomalies, boundary that divides significantly different anomalies on both sides, sudden widening and narrowing parts of closed anomaly contours, and isomorphically distorted parts of the contours.^42^ The electrical anomalies differ greatly on both sides of the faults on the resistivity profile. The existence of the fault is reflected by the high- and low-resistivity anomalies, the dense transition zones between vertical high and low anomalies on resistivity contours, and vertical dislocation of low-value anomalies of phases.^27^ Signs indicating the presence of faults on the seismic profiles include dislocation or deflection of reflection events; bifurcation, merging, deflection, or strong phase-conversion of reflection events; sudden increases, decreases, or disappearance of reflection events; sudden variation of interval of reflection sequences; sudden variation of occurrence of reflection events; and disordered reflections or appearance of a blank zone.^44^

### 4.2 Developmental Characteristics of Faults

**Figure 10:**
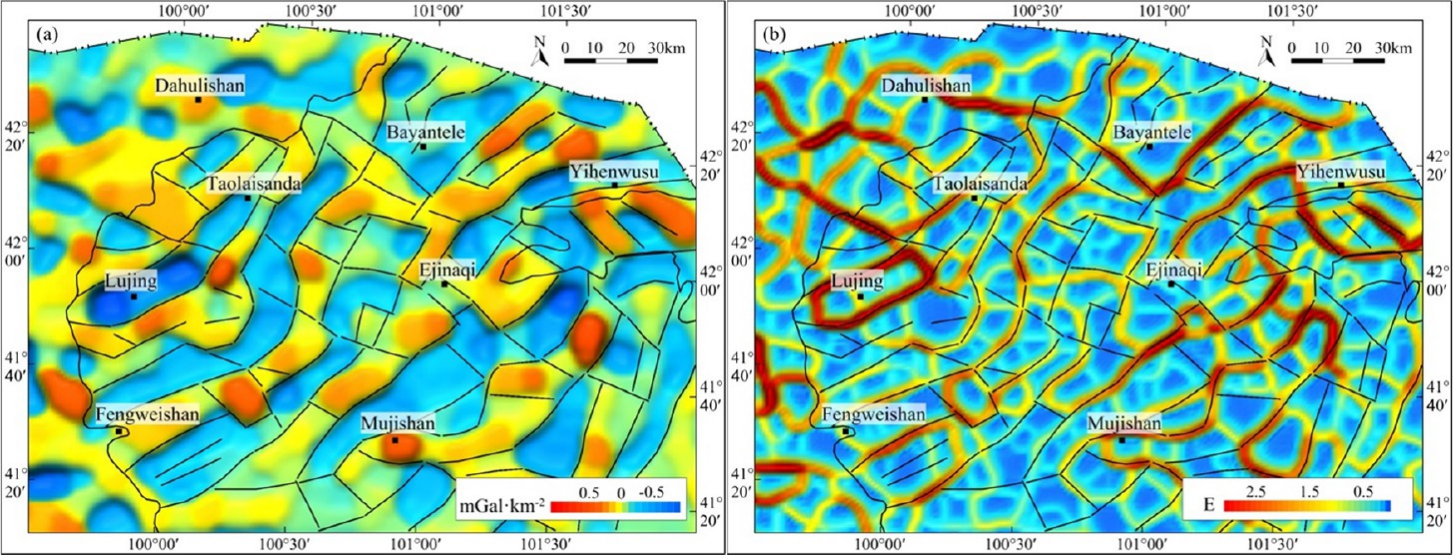
(a) Vertical second derivative gravity anomaly and the fault system. (b) Total horizontal derivative gravity anomaly and the fault system.

**Figure 11:**
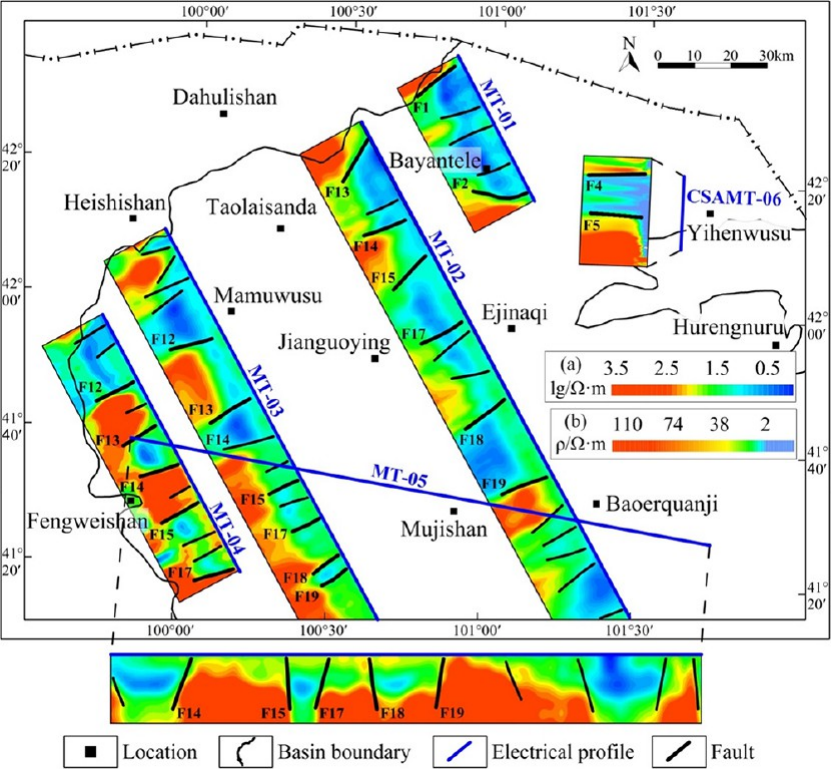
Electrical profiles and their interpretations of faults. Color bar legend (a) is for MT-01, MT-02, MT-03, MT-04, and MT-05 profiles; color bar legend (b) is for the CSAMT-06 profile. MT-02 adapted with permission from ref (3). Copyright [Geological Publishing House, 2012]. MT-03 adapted with permission from refs (8,27). Copyright [Geological Bulletin of China, 2011] and [Geological Bulletin of China, 2011]. MT-05 adapted with permission from ref (40). Copyright [Geological Bulletin of China, 2014]. CSAMT-06 adapted with permission from ref (30). Copyright [Computing Techniques for Geophysical and Geochemical Exploration, 2022].

**Figure 12:**
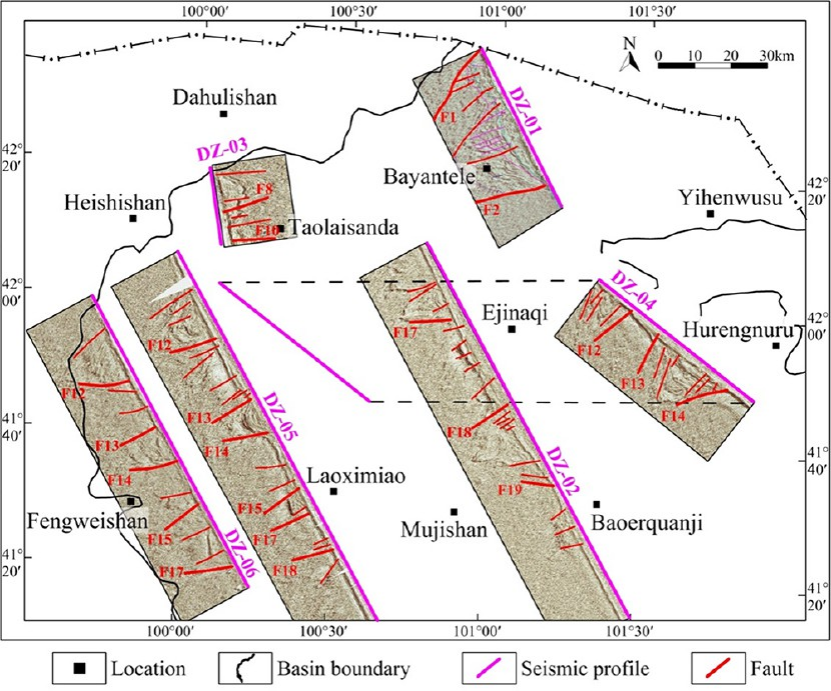
Seismic profiles and their interpretations of faults. DZ-01 adapted with permission from ref (10). Copyright [School of Geoscience Yangtze University, 2013]. DZ-02 adapted with permission from refs (10,36). Copyright [School of Geoscience Yangtze University, 2013] and [Journal of Geophysics and Engineering, 2024]. DZ-04 adapted with permission from ref (10). Copyright [School of Geoscience Yangtze University, 2013].

**Figure 13:**
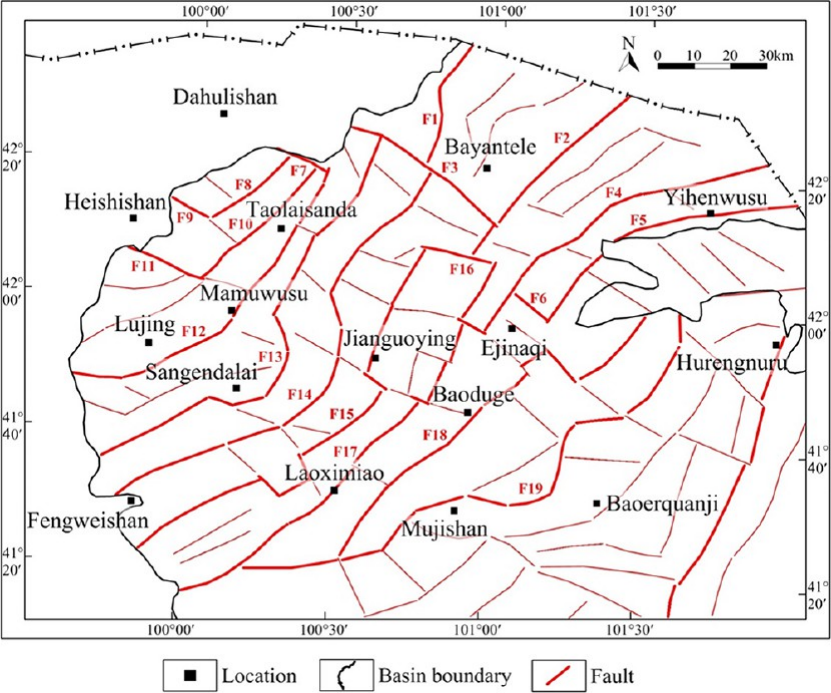
Distribution map of fault system in the Juyanhai Depression.

Since sedimentation of the Carboniferous-Permian system, the research region had experienced tectonic reworking in the Late Variscan, Early-Mid Yanshanian, Late Yanshanian, and Himalayan periods, forming complex fault systems.^2,45^ Mainly based on a gravity anomaly (Figure 10), the distribution of fault systems in the Juyanhai Depression was determined by comparing, analyzing, and interpreting electrical and seismic profiles in conjunction with previous research results (Figures 11–13).

Faults that control the basement structure or control boundaries of Mesozoic tectonic units were classified as primary faults; faults that control the distribution of sedimentary strata in tectonic units and exert key influences on tectonic units were classified as secondary. In this way, 19 primary faults and several secondary faults were classified in the area of interest (Figure 13). Therein, primary faults are main faults that control the formation and distribution of structures, such as sags and arches (Table 1); secondary faults are mainly located inside sags or arches and further divide the sags and arches into multiple secondary structures.

**Table 1:** Statistical Table of Fault System in the Juyanhai Depression

fault no.	fault name	strike	dip	fault length/km	geological significance
F1	Judong No.1 fault	NNE	SEE	36	Boundary control fault on the west side of the Judong sag
F2	Judong No.2 fault	NE	NW	50	Boundary control fault on the east side of the Judong sag
F3	Judong No.3 fault	NW	NE	50	Boundary control fault on the south side of the Judong sag
F4	Yihenwusu west fault	NE	SE	94	Boundary control fault on the west side of the Yihenwusu sag
F5	Yihenwusu east fault	NE	NW	81	Boundary control fault on the east side of the Yihenwusu sag
F6	Yihenwusu south fault	NW	NE	11	Boundary control fault on the south side of the Yihenwusu sag
F7	Wuzhuer north fault	near EW	near S	15	Boundary control fault on the north side of the Wuzhuer sag
F8	Wuzhuer east fault	NE	NW	27	Boundary control fault on the east side of the Wuzhuer sag
F9	Wuzhuer south fault	NWW	NNE	11	Boundary control fault on the south side of the Wuzhuer sag
F10	Lujing west fault	NNE	SEE	44	Boundary control fault on the west side of the Lujing sag
F11	Lujing north fault	NWW	SSW	23	Boundary control fault on the northwest side of the Lujing sag
F12	Lujing east fault	NNE-NE	NWW-NW	99	Boundary control fault on the east side of the Lujing sag
F13	Tiancao west fault	NNE-SN-NE	SEE-E-SE	134	Boundary control fault on the west side of the Tiancao sag
F14	Tiancao east fault	NNE-SN-NE	NWW-W-NW	137	Boundary control fault on the east side of the Tiancao sag
F15	Gelangwusu west fault	NNE-NE	SEE-SE	138	Boundary control fault on the west side of the Gelangwusu sag
F16	Gelangwusu north fault	near EW	near S	20	Boundary control fault on the north side of the Gelangwusu sag
F17	Gelangwusu east fault	NNE-NE	NWW-NW	130	Boundary control fault on the east side of the Gelangwusu sag
F18	Jigeda west fault	NE	SE	122	Boundary control fault on the west side of the Jigeda sag
F19	Jigeda east fault	NE-NEE	NW-NNW	161	Boundary control fault on the east side of the Jigeda sag

According to the fault strike, three groups of faults, namely, the NE (or NNE)-, NW (or NWW)-, and near-EW-trending faults, are developed in the research region. With regard to the number of faults developed, faults in the region are mainly NE (or NNE)-trending, followed by NW (or NWW)-trended, while there are a small number of near-EW-trending faults. In terms of the fault scale, primary faults extend to a long distance on the plane and cut strata deeply along the vertical direction, which are mainly dominated by NE (or NNE)-trended faults, accompanied by a few NW (or NWW)- and near-EW-trending ones. Secondary faults extend to a short distance on the plane and cut strata shallowly vertically. They are mainly dominated by NW (or NWW)- and near-EW-trending faults, with a few NE (or NNE)-trending ones.

From the perspective of overall distribution characteristics, faults in the north (north to the Mamuwusu-Jianguoying-Baoduge line) and east (east to the Baoerquanji-Hurengnuru line) of the research region are mainly NNE-trending. Faults in the south (south of the Mamuwusu-Jianguoying-Baoduge line) of the region are mainly NE-trending. Faults in the center (north and south around the Heishishan-Mamuwusu-Jianguoying-Baoduge line) and northwest (F3 and faults nearby) of the region are mainly NW (or NWW)-trended. Faults near Mujishan in the south and F16 in the north of the region are nearly EW-trended. The figure shows that the region has a small number of nearly EW-trended faults, and it is mainly dominated by NE (or NNE)- and NW (or NWW)-trended faults (Figures 10 and 13). The NE (or NNE)- and NW (or NWW)-trending faults in the center (along the Mamuwusu-Jianguoying-Baoduge line) cut each other and are distributed in an S-shaped en-echelon pattern on the plane.

Interpretation results on electrical and seismic profiles indicate that faults in the research region are all normal faults and are steep in the upper part while gentle in the lower part of the profiles. Main faults were mostly developed in the Early Yanshanian period and ended in the Late Cretaceous period, and some faulting activities lasted until the Himalayan period. Faulting activities determine the local micromorphologies of sedimentary facies in spatial distribution and also lead to the difference in thicknesses of the same stratigraphic unit on each side of a fault to different extents.^2^ They play an important role in controlling the formation of local structures and hydrocarbon accumulation.

## 5 Results

### 5.1 Division Scheme of Tectonic Units

**Figure 14:**
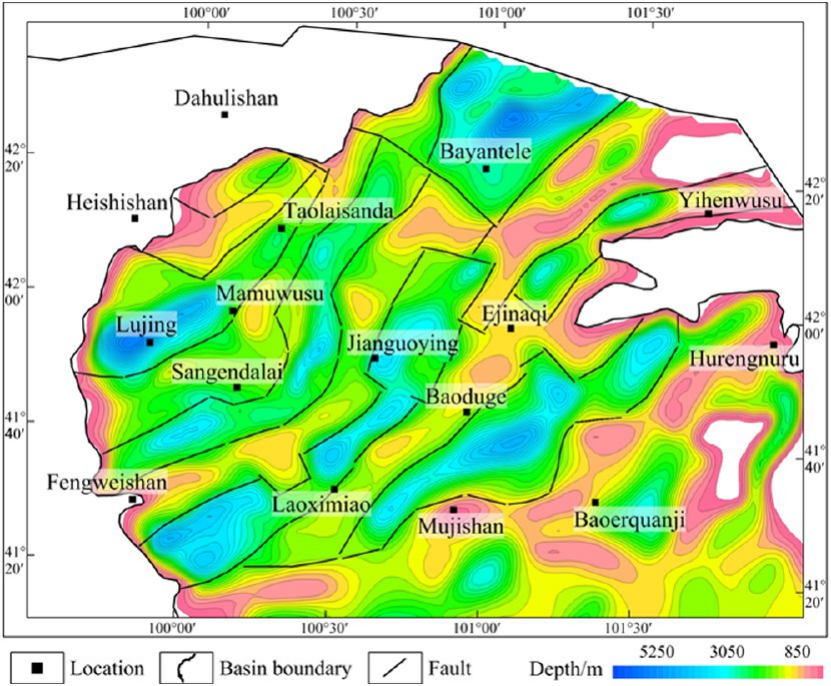
Burial depth map of sedimentary strata in the Juyanhai Depression. Adapted with permission from ref (36). Copyright [Journal of Geophysics and Engineering, 2024].

**Figure 15:**
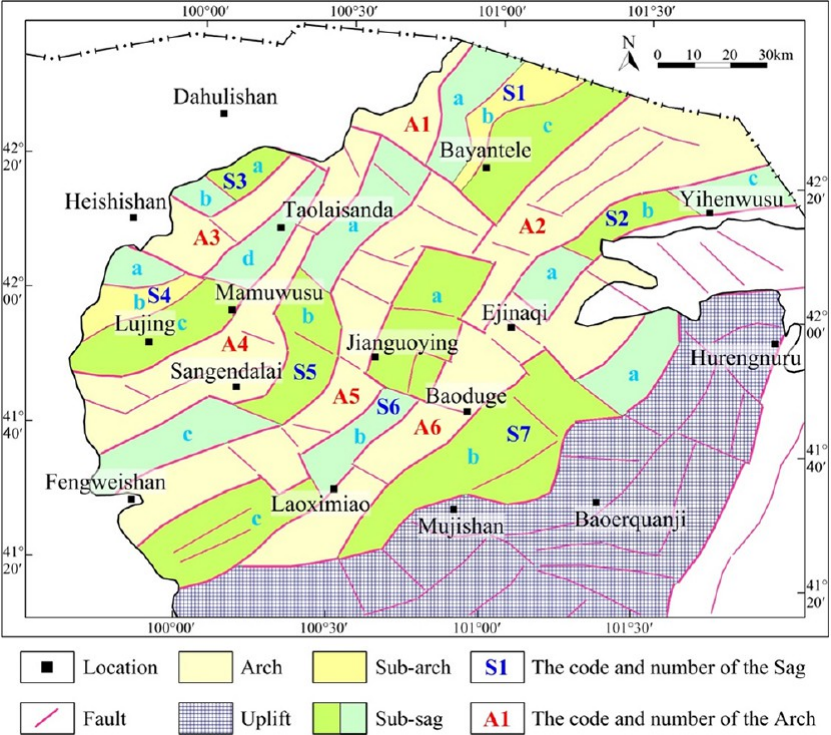
Distribution map of current structural units in the Juyanhai Depression.

Combining with interpretation results of electrical and seismic profiling data and referring to distribution of fault systems in the region (Figures 11–13), and considering factors including the burial depth of sedimentary strata (Figure 14) and surface outcrop (Figure 12), the Juyanhai Depression could be divided into 13 primary structures (Figure 15; six arches, and seven sags). According to anomaly characteristics on maps of residual gravity anomalies, gravity anomalies obtained by VSD, THD of gravity anomalies (Figures 7, 8, 10). The seven sags were further classified into 20 secondary structures.

### 5.2 Distribution Characteristics of Tectonic Units

Tectonic units divided in the research mainly reflect the existing tectonic pattern of the Juyanhai Depression and reveal distribution characteristics of Mesozoic structures in the region. Compared with previous division results,^2−4^ the boundary locations and distribution ranges of tectonic units were further determined via combined interpretation of gravity, electrical, and seismic data and secondary structures were further divided within the sags. According to the division scheme of tectonic units, the Juyanhai Depression covers a total area of 17,454 km^2^, in which the total area of six arches is 8080 km^2^ (Table 2, 46.3% of the total area); the area of the seven sags is 9374 km^2^ (Table 3, 53.7% of the total area).

**Table 2:** Statistical Table of Arches in the Juyanhai Depression

no.	tectonic unit	area/km^2^	maximum buried depth of basement/m
A1	Juxi arch	333	2000
A2	Bulongtu arch	1755	1500
A3	Lubei arch	565	1250
A4	Lunan arch	1566	2000
A5	Wujiajing arch	1977	1700
A6	Baoduge arch	1884	1500

**Table 3:** Statistical Table of Sags in the Juyanhai Depression

no.	tectonic unit	sub no.	sub unit	area/km^2^	maximum buried depth of basement/m
S1	Judong sag	S1a	Western subsag	418	4000
		S1b	Middle subarch	326	3200
		S1c	Eastern subsag	709	5500
S2	Yihenwusu sag	S2a	Yinan subsag	234	2800
		S2b	Yizhong subsag	279	2700
		S2c	Yibei subsag	286	1700
S3	Wuzhuer sag	S3a	Wubei subsag	170	2200
		S3b	Wunan subsag	110	1100
S4	Lujing sag	S4a	Lujingbei subsag	175	1650
		S4b	Lujingbei subarch	257	2000
		S4c	Mamuwusu subsag	626	5500
		S 4d	Taolaisanda subsag	339	2500
S5	Tiancao sag	S5a	Tianbei subsag	553	3000
		S5b	Tianzhong subsag	583	3700
		S5c	Tiannan subsag	535	3500
S6	Gelangwusu sag	S6a	Jianguoying subsag	805	3400
		S6b	Laoximiaobei subsag	339	3900
		S6c	Laoximiaonan subsag	780	4400
S7	Jigeda sag	S7a	Jibei subsag	417	3800
		S7b	Jinan subsag	1433	4500

With regard to the strike of structures, sags and arches in the research region are distributed alternately in rows and long strips along the NE (or NNE) direction on the whole. In addition, sags and arches are independent and separated from each other.^2^

From the perspective of anomaly characteristics, on the residual gravity anomaly and the VSD gravity anomaly maps, the sag areas generally show low gravity anomalies, while the arch areas generally exhibit high gravity anomalies (Figure 7e,f). On electrical profiles, the sags are generally low-resistivity zones, while the arches are high-resistivity zones (Figure 11). Different structures differ more remarkably in terms of the seismic profiles. Reflection sequences are developed in sags, with well-developed reflection sequences in the shallow, middle, and deep parts of profiles and a high SNR. Arches have a low SNR, and the middle and deep parts show chaotic reflection sequences or do not contain effective reflection sequences (Figure 12).

With regard to the area of tectonic units and burial depth of the basement, Juxi and Lubei arches in the northwest of the region cover small areas, which are generally 300–600 km^2^, while the areas of four arches (Bulongtu, Lunan, Wujiajing, and Baoduge) in the central and eastern parts are large, reaching 1500–2000 km^2^; the maximum burial depth of the basement in arch areas is generally larger than 1000 m while being smaller than 2000 m. Wuzhuer and Yihenwusu sags in the north of the region cover small areas, which are both smaller than 800 km^2^; the remaining five sags (Judong, Lujing, Tiancao, Gelangwusu, and Jigeda) cover large areas (1300–2000 km^2^); the maximum burial depth of the basement in sag areas is generally larger than 1600 m and can reach 5500 m at most.

From the perspective of stratigraphic distribution, drilling data show that the strata developed in sags mainly include Quaternary Holocene series, Suhongtu and Bayingebi formations of the Mesozoic lower Cretaceous series, the upper Permian, middle-lower Permian, and lower Permian-upper Carboniferous Ganquan formation of the upper Palaeozoic strata. Most of the drilled wells were completed in the Ganquan formation (not drilled through), and individual wells were completed after encountering intrusive rocks.^21^ In comparison, drilling data of arch areas unveil a significant difference in the strata. Multiple wells were directly drilled to Permian-upper Carboniferous Ganquan formation after drilling through Cenozoic strata; therefore, Mesozoic strata are absent (or Mesozoic strata are thin), and the thickness of the Ganquan formation varies greatly. Altered andesite basalt in the Devonian system or middle Varissian intrusive rocks were encountered in several wells after drilling through the Ganquan formation.^18^

## 6 Discussion

### 6.1 Overview of Multiple Resource Exploration

**Figure 16:**
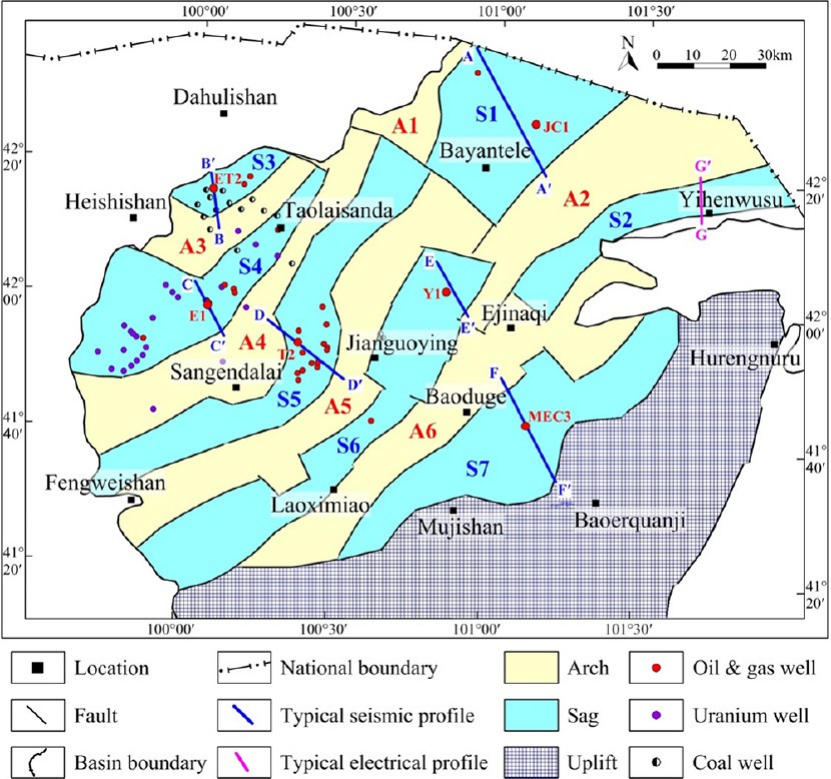
Map of current structural units and distributions of different types of wells in the Juyanhai Depression.

In order to investigate and explore multiple resources, based on existing data statistics, a total of 72 wells of various types have been constructed in different tectonic units in the Juyanhai Depression (Figure 16, Table 4). Multiple wells, including well E1, well T6, and well MEC3, are found to have industrial oil–gas flow. Drilling of well MEC1, well Y1, and well JC1 shows good oil-gas indication, suggesting favorable oil–gas exploration prospect in the region.^5,12,15,24,46−49^ Favorable shale oil and gas indication is found in geological survey wells including well ET2 and coal well 0–1, indicative of good prospects of shale oil and gas in the region.^16^ Drilling data of wells including zk1 demonstrate that 2.1 minable coal seams are encountered in a single well on average in the research region, and the average thickness of minable coal seams in a single well is 4.77 m, indicating a favorable prospect of coal resources.^18^ The uranium mineralization clue is found in ZK8–2 and other wells, verifying uranium mineralization potential and prospecting space in the region.^20^ Comprehensive analysis shows that the Juyanhai Depression has abundant resources, including oil and gas, shale oil and gas, coal, and uranium, and favorable exploration prospects for multiple types of resources.

**Table 4:** Statistical Table of Various Types of Wells in the Juyanhai Depression

no.	tectonic unit	wells	type
S1	Judong sag	JC1, MED2	Oil-gas wells^16,21^
S3	Wuzhuer sag	MEC1, MED1, ET2	
		zk1, Yb-4, zk902, 0–1, 0–2, 15–1	Coal wells^18^
A3	Lubei arch	zk901, zk903, Yb-1, Yb-2, Yb-3	
		ZK0–1	Uranium well^20^
S4	Lujing sag	zk905, zk906	Coal wells^18^
		E1, E4, ET1, ET3, XT1, XT4, XT5, XT8, XT9, XT10, L1	Oil-gas wells^21,47^
		ZK0–2, ZK0–3, ZK1–1, ZK1–2, ZK1–3, ZK3–1, ZK3–2, ZK3–3, ZK3–4, ZK3–5, ZK4–1, ZK5–1, ZK5–2, ZK8–1, ZK8–2, ZK110, ZKL10, ZKL20, ZKL30	Uranium well^20^
A4	Lunan arch	ZKL11, ZKL21, ZK4–2	
		zk904	Coal well^18^
S5	Tiancao sag	T1, T2, T3, T5, T6, T601, T602, T603, T7, T701, T702, T8, T9, T10	Oil-gas wells^21,24^
S6	Gelangwusu sag	zk907, zk908	Coal wells^18^
		Y1, MEC2	Oil-gas wells^21^
S7	Jigeda sag	MEC3	

### 6.2 Structural Characteristics of Sags

**Figure 17:**
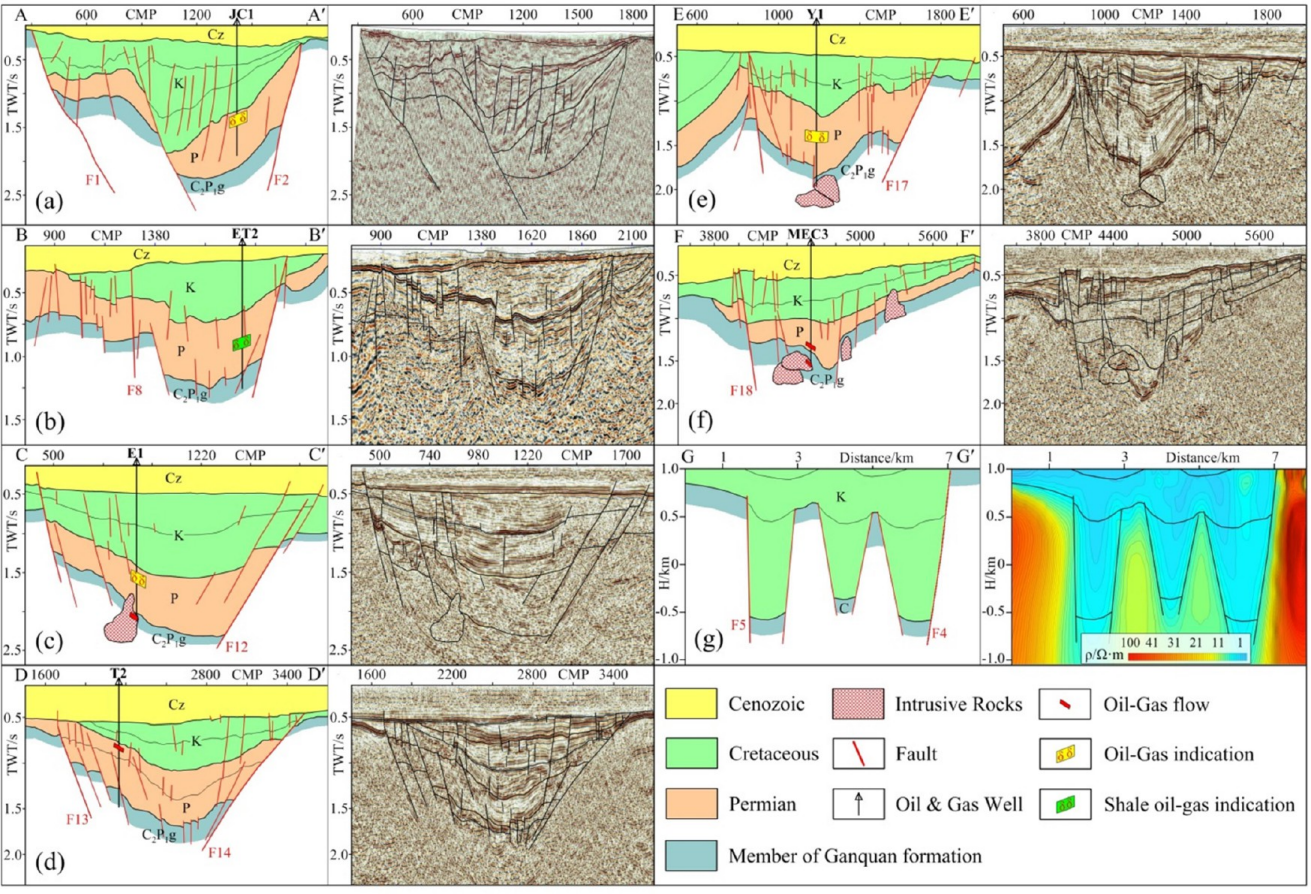
Geological structure map of each sag in the Juyanhai Depression. (a) Judong sag. Adapted with permission from ref (10). Copyright [School of Geoscience Yangtze University, 2013]. (b) Wuzhuer sag. (c) Lujing sag. (d) Tiancao sag. (e) Gelangwusu sag. (f) Jigeda sag. Adapted with permission from ref (36). Copyright [Journal of Geophysics and Engineering, 2024]. (g) Yihenwusu sag. Adapted with permission from refs (30,50). Copyright [Computing Techniques for Geophysical and Geochemical Exploration, 2022] and [Geological Bulletin of China, 2023].

According to locations of wells (Figure 16, Table 4), planar distribution of existing wells is closely related to existing tectonic units: oil–gas wells are all distributed in sags, coal wells are mainly distributed in sags and arches from the north to the Heishishan-Jianguoying line to Taolaisanda, while uranium wells are concentrated in the Lujing sag and adjacent arch structures. Statistical results in Figure 16 and Table 4 show that most wells are mainly distributed in the sags. In a new round of resource potential evaluation, 27 sags favorable for resource exploration were selected in the Yingen-Ejinaqi Basin as the next key evaluation zones.^15^ The seven sags, including Tiancao sag in the Juyanhai Depression, are considered to have favorable exploration prospects. The further to characterize geological structures in sags, six typical seismic profiles around key wells and one typical electrical profile were selected and analyzed according to the intensity of exploration amount of data collection from various sags (Figure 17).

According to geological profiles interpreted by seismic and electrical data of each sag in the Juyanhai Depression, sags in the research region are divided into single- and double-fault ones by analysis from the perspective of structural patterns of sags. Among all sags, Judong, Wuzhuer, and Jigeda sags belong to single-fault ones; Lujing, Tiancao, Gelangwusu, and Yihenwusu sags are double-fault features.^2,4,51,52^

Single-fault sags are characterized by control of their formation by boundary-controlling normal faults and are shown as half-graben fault depressions, with the subsidence center closely adjacent to the downthrown side of controlling faults. They can be further divided into single-fault bench-type, single-fault trough-type, and single-fault overlap-type features according to the degree of fault development, internal structure, and stratigraphic overlap relationship.

Double-fault sags are characterized by control of their formation by means of normal faults on both sides, while the fault displacements of sag-controlling faults differ on both sides. The main subsidence center is in close vicinity of the downthrown side of controlling faults. In accordance with the development degree of secondary fault, internal structure, and stratigraphic relationship, the double-fault sags in the region are all double-fault graben-type features.^51^

### 6.3 Oil-Gas Favorable Exploration Areas

According to characteristics of seismic data and combined with previous research, the sags and favorable oil–gas exploration areas can be analyzed as follows:

1. Single-fault bench-type sags, such as the Judong sag, develop several faults showing the same dip direction with the controlling fault in the steep slope and form a central fault zone near the secondary subsags, thus forming fault noses and fault-block traps. They develop several normal faults showing the opposite dip direction to that of the boundary fault in the gentle slope, of which the fault displacement is generally so small that they do not control the development of fault troughs together with the boundary fault. The fault blocks and fault noses developed near subsags and various fault-screened traps developed in the gentle slope provide favorable conditions for petroleum entrapment and represent the most favorable structures for exploration near source rocks.
2. Single-fault trough-type sags, such as the Wuzhuer sag, develop several normal faults with the dip direction opposite to that of the boundary fault in the gentle slope. Those adjacent to the secondary subsags are secondary normal faults, which control the development of fault troughs together with the boundary normal fault and forming the main oil-generating subsag; the multistage fault zones in the slope can form fault-block traps. Therein, the multistage normal faults developed in the gentle slope form fault noses and fault-block traps. These structures are in the close vicinity of the center of source rocks in the secondary subsag and represent the favorable exploration targets.^51^
3. Single-fault overlap-type sags, such as the Jigeda sag, maintain the overlap structure in the complete development process of rift depressions, are characterized by overlap of strata toward the gentle slope, and pinch-out in the up-dip direction. Structural traps are undeveloped in the slope, and lithostratigraphic traps are emphases of exploration. Fault noses and fault-block traps developed in the multistage step-fault zone in the gentle slope are near to source rocks in the secondary subsag, which exhibit the most favorable reservoir-forming conditions within the effective range of hydrocarbon supply.
4. The structural development in double-fault graben-type sags, such as the Lujing, Tiancao, Gelangwusu, and Yihenwusu sags, shares similar characteristics to single-fault sags in terms of the main fault zone that controls subsidence and secondary sag-controlling faults show large subsidence. Therefore, rollover anticlines and fault-block traps are developed in the subsidence-controlling fault zones, and secondary faults in the sags do not control anticlines any longer while mainly controlling the sedimentation center of fault troughs instead. Fault-nose traps in subsecondary fault zones near sags are more favorable for capturing oil and gas.^51^

The research presumes that faults in the Juyanhai Depression control the development pattern of tectonic units and also sedimentation, while fault-screened traps formed due to fault screening of the up-dip direction of strata are structural traps most developed in the region.^2^ Despite inferior conditions to anticlinal traps, fault-screened traps have a large number, and some are also at locations favorable for oil–gas migration. Therefore, fault-screened traps in the research region are key to study of the structural traps and also among the most important drilling targets, especially fault-block traps in slopes and gentle slopes in the sags, which are the first choice for drilling.^2,53^

The basin simulation results of typical sags in the Yingen-Ejinaqi Basin show that different sags exhibit their own hydrocarbon accumulation characteristics^2^; however, hydrocarbon-generating sags with same structures developed in a similar tectonic setting follow similar trends of oil–gas migration and accumulation. Industrial oil–gas flow is found during drilling in multiple sags in the research region. To be specific, light crude of 2.52 m^3^/day and natural gas of 1902 m^3^/day are obtained in late Carboniferous intrusive rocks during formation testing of well E1 in Lujing sag^21^; high-yield industrial oil flow with the daily production of 20 m^3^ is achieved in well T6 in Tiancao sag^5^; light crude of 2.64 and 1.66 m^3^/day is drilled separately from late Carboniferous intrusive rocks and middle-lower Permian sandstone from well MEC3 in the Jigeda sag.^21^ Favorable oil–gas indication is found in multiple wells, including well Y1 in the Gelangwusu sag, well MEC1 in the Wuzhuer sag, and well JC1 in the Judong sag.

Analysis indicates that oil and gas migrate and accumulate in the research region, with sags as the independent units, forming relatively independent petroleum systems or exploration units, that is, a sag is a complete petroleum system. Drilling data of multiple wells show that oil–gas reservoirs related to intrusive rocks are developed in the research region; therefore, intrusive rocks are important oil–gas exploration areas in the region. Generally, sags in the research region are small, source rocks are mainly distributed in the near-slope zones of single-fault sags and in the side near to secondary faults of double-fault sags, and oil–gas migration and accumulation mainly occur along vertically, supplemented by lateral migration. Because the sedimentation center of sags overlaps the subsidence center, hydrocarbon generation, migration, and accumulation occur along the up-dip direction of the short axis that is vertical to the sag strike at the center of hydrocarbon-generating deep sags. Once there is a fault-screened trap, lithologic trap, or unconformity trap above the up-dip direction, hydrocarbon is accumulated.^2^

## 7 Conclusions

1. The combined processing and interpreting results of gravity anomalies as well as electrical and seismic profiles of the Juyanhai Depression can reveal the distribution characteristics of fault systems in the research region and accurately characterize the development of tectonic units in the region. Therein, the filtering enhancement results of the VSD gravity anomaly and the total horizontal gradient anomaly after small subdomain filtering exhibit favorable application effects in tectonic division and fault identification.
2. The Juyanhai Depression is affected by the development of three sets of faults: the NE (or NNE)-, NW (or NWW)-, and near-EW-trending faults. Among them, the main faults are NE (or NNE)-trending features, followed by NW (or NWW)-trending ones, while there are fewer near-EW-trending ones. Faults in the region of interest are all normal faults that are steep in the upper part and gentle in the lower part of the profile. Faulting activities not only lead to the thicknesses difference of sedimentary strata on both sides of faults but also play an important role in controlling the formation of local structures and hydrocarbon accumulation.
3. The Juyanhai Depression can be divided into six arches and seven sags. The sags and arches are generally distributed in a NE (or NNE) direction in rows, with narrow elongated strips distributed alternately. The sags and arches are independent and separated from each other. Strata are well-developed in sags and the maximum burial depth of basement is generally larger than 1600 m and can reach 5500 m at most. Mesozoic strata are absent or thin in arch areas, where the maximum burial depth of basement is generally larger than 1000 m while being smaller than 2000 m.
4. Four types of sags (single-fault bench-type, single-fault trough-type, single-fault overlap-type, and double-fault graben-type features) are developed in the Juyanhai Depression. Oil–gas exploration should mainly focus on sags, mainly be directed toward igneous bodies or bedrock buried hills in the deep part while focusing on slopes in the shallow part. Specifically, exploration of oil–gas reservoirs should be based on fault-anticlines, fault blocks, fault-screened traps, and lithologic oil–gas reservoirs, while also considering exploration in igneous bodies or bedrock buried hills.

## References

[ref1] Bai Y. The tectonic characteristics and oil-gas bearing prospects of the west Inner Mongolia basin. J. Xi’an Pet. Inst. 1996, 11, 15–18.

[ref2] Wei P.; Zhang H.; Chen Q.; Guo Y.; He H.; Wang X.; Li B.; Lin W.; Zhang J.; Li T. Oil and Gas Geological Characteristics and Exploration Prospects in Yingen-Ejinaqi Basin; Petroleum Industry Press, 2006.

[ref3] Lu J.; Chen G.; Li Y.; Wei X.; Wei J.; Jiang T.; Shi J.; Dang B.; Zhao X.; Liu J. Geological Conditions and Resource Prospects of Carboniferous-Permian Oil and Gas in the Yin’e basin and Its Adjacent Areas; Geological Publishing House, 2012.

[ref4] Li G.; Qi W.; Tang L.; Fan T. Prospects for Oil and Gas Exploration in Juyanhai Depression, Yin’e Basin. J. Oil Gas Technol. 2007, 29 (5), 13–18.

[ref5] Li T.; Huang Z.; Yin Y.; Gou H.; Zhang P. Sedimentology and geochemistry of Cretaceous source rocks from the Tiancao Sag, Yin’e Basin, North China: implications for the enrichment mechanism of organic matters in small lacustrine rift basins. Journal of Asian Earth Sciences. 2020, 204, 104575 10.1016/j.jseaes.2020.104575.

[ref6] Wei X.; Lu J.; Wei J.; Xu H.; Li Y. The revision of stratigraphic division of X well in Juyanhai depression, Yingen-Ejin Banner basin, Inner Mongolia, and its geological significance. Geol. Bull. China 2014, 33 (9), 1409–1416.

[ref7] Zhang J.; Li F.; Sun B.; Yang S.; Zhang B.; Ma Y. Formation classification of Juyanhai depression in Yin-E basin. Oil Geophys. Prospect. 2020, 55 (4), 892–897.

[ref8] Yan Y.; Yuan B.; Yang G.; Zhang C.; Shen A.; Xu H. The characteristics of gravity field and fault structure in Yingen-Ejinaqi basin, western Inner Mongolia. Geol. Bull. China 2011, 30 (12), 1962–1968.

[ref9] Xiong S.; Zhou D.; Ding Y.; Guo Z.; Li Z.; Liang X.; Xiao M.; Duan H.; Hu Y.; Tong J. Atlas of Oil and Gas Prospective Evaluation of Yin’e-Hexi corridor Basin; Geological Publishing House, 2020.

[ref10] Huang H. Tectonic framework and regional evolution characteristics of the upper palaeozoic in Yin-E basin - A case study in Juyanhai depression; Ph.M. Thesis, School of Geoscience Yangtze University: Hubei, 2013.

[ref11] Jin J.; Meng Q.; Zhang Y.; Xu D. Evolution and hydrocarbon features of the Jurassic and Cretaceous basins, Ejinaqi. Acta Petrol. Sin. 2000, 21 (4), 13–19.

[ref12] Lu J.; Zhang H.; Niu Y.; Liu H.; Chen Q.; Wei J. Carboniferous-Permian petroleum conditions and exploration breakthrough in the Yingen-Ejin Basin in Inner Mongolia. Geol. China 2017, 44 (1), 13–32. 10.12029/gc20170102.

[ref13] Wei P.; Zhang H.; Lin W.; Li A. Exploration prospects for oil and gas in Yingen-Ejinaqi Basin. Nat. Gas Ind. 2005, 25 (3), 7–10.

[ref14] Wei J.; Zhao Y.; Zhou J.; Zhang Y.; Wang L.; Jiang T.; Wang B. Geochemical characteristics of crude oil in Jigeda sag of Yingen-Ejin Basin and its significances. NW Geol. 2023, 56 (5), 332–342.

[ref15] Zhang H.; Li L.; Chen Q.; Wan J.; Shi D.; Peng G.; Zhou Y. New field, new types and resource potentials of oil-gas exploration in Yin’gen-Ejinaqi Basin. Acta Petrol. Sin. 2024, 45 (1), 147–162. 10.7623/syxb202401009.

[ref16] Wei J.; Wang B.; Qiao S.; Zhang H. A preliminary study on the geological conditions of the Permian shale oil (gas) in Xirehada area, western Inner Mongolia. NW Geol. 2018, 51 (3), 200–213.

[ref17] Zhang H.; Li J.; Wang X.; Shi D.; Chen Q.; Fan X.; Si M.; Zhao Q. Formation and evolution of Yin’gen-E’ji’naqi basin and prospects for oil and gas exploration. Pet. Geol. Exp. 2020, 42 (5), 780–789. 10.11781/sysydz202005780.

[ref18] Lu J.; Wei X.; Wei J.; Jiang T.; Niu Y.; Tao J.; Xu Y. The Carboniferous-Permian coal-bearing bed in Saihan Toroi area of Ejin Banner within Yin’e basin, Inner Mongolia, and its significances. Geol. Bull. China 2013, 32 (10), 1653–1664.

[ref19] Yang Z.; Liu W.; Niu T.; Ji H.; Zhang W.; Li W. Early cretaceous sedimentary evolution of Juyanhai depression in Yin’e basin and its influence on the formation of sandstone-type Uranium deposits. World Nucl. Geosci. 2023, 40 (2), 162–173.

[ref20] Luo S.; Niu T.; Meng L.; Wang G.; Liu W.; Ji H.; Meng Q. Analysis of Uranium Metallogenic Conditions and Prospection Direction in Lujing Sag, Badan Jaran Basin. Uranium Geol. 2024, 40 (1), 157–170. 10.3969/j.issn.1000-0658.2024.40.013.

[ref21] Lu J.; Wei J.; Jiang T.; Wang B.; Gou H.; Wang J.; Shang Y.; Xu H. Stratigraphic correlation of the wells of Juyanhai depression of Yingen-Ejin Basin and age constraints on oil and gas layers. Geol. China 2023, 50 (5), 1311–1326. 10.12029/gc20200107001.

[ref22] Lin W.; Zhou Y.; Wang X.; Zhang H.; Wang H. Structural-depositional System and Factors Affecting the Hydrocarbon Pool Formation in Tiancao Depression, the Yingen-Ejinaqi Basin. Geotecton. Metallog. 2004, 28 (4), 444–449.

[ref23] Li G. Structural feature of Lujing depression and analysis of oil/gas prospect. Oil Geophys. Prospect. 2004, 39 (4), 443–449.

[ref24] Long F.; Wang J.; Wen Y.; Hu J.; Gou H. Integrated seismic data processing & interpretation applied in Tiancao Sag. Oil Geophys. Prospect. 2018, 53 (S2), 251–255.

[ref25] Zhang C.; Yang G.; Yan Y.; Yuan B.; Xu H. Features of aeromagnetic anomalies in Yingen-Ejin Banner basin and their geological significance. Geol. Bull. China 2012, 31 (10), 1724–1730.

[ref26] Liu J.; Li X.; Zhang Q. The application of Gravity-Magnetic-Magnetotelluric Joint Inversion to the Quantitative Interpretation of Yin’E Basin. Geophys. Geochem. Explor. 2013, 37 (5), 853–858.

[ref27] Liu J.; Shen A.; Chen X. Application of magnetotelluric sounding for Carboniferous-Permian petroleum geological survey in Yingen-Ejin Banner basin, western Inner Mongolia. Geol. Bull. China 2011, 30 (6), 993–1000.

[ref28] He M.; Zhang Y.; Pei F.; Zhang X.; Wang X.; Li Y. Electrical characteristics of Carboniferous-Permain mudstone beds in Juyanhai Depression, Yingen-Ejin Basin. Geophys. Prospect. Petrol. 2022, 61 (5), 929–939. 10.3969/j.issn.1000-1441.2022.05.017.

[ref29] Xu H.; Wang B.; Zhou J.; Jiang T.; Han X.; Zhao F.; Yuan B.; Ma J. Research on the method of gravity data edge expanding based on step by step iterative interpolation. NW Geol. 2023, 56 (2), 306–321.

[ref30] Wang W.; Lei C.; Bo H.; Wang Z.; Yang B.; Xu H.; Zhao X.; Yang K. Application of composite geophysical exploration method in 1:50000 regional geological survey in shallow coverage area—taking Ejina banner area as an example. Comput. Tech. Geophys. Geochem. Explor. 2022, 44 (2), 235–244.

[ref31] Gou L.; Zhang Z.; Zeng X.; Li C.; Zhang Y.; Yan L. Wavefield characteristics of common-scatterpoint gathers and their application to seismic processing and interpretation in the Yin’e Basin. Geophysics. 2023, 88 (2), B55–B68. 10.1190/geo2022-0249.1.

[ref32] Duan R.; Li X.; Liu J.; Guo C.; Qiang Y.; Liu J. Distribution of magmatic rocks in eastern Yin’e basin revealed by 3D gravity and magnetic inversion. J. Appl. Geophys. 2023, 215, 105068 10.1016/j.jappgeo.2023.105068.

[ref33] Zhang P.; Fang H.; Zhang X.; Zhang Y.; Xu H.; Peng Y.; Yuan Y. Magnetotelluric sounding evidence of development of nappes in the Tuolai Sag, Yin-E basin. Acta Geophys. 2020, 68 (1), 91–104. 10.1007/s11600-019-00378-z.

[ref34] Gupta V.; Ramani N. Some aspects of regional-residual separation of gravity anomalies in a Precambrian. Geophysics. 1980, 45, 1412–1426. 10.1190/1.1441130.

[ref35] Jacobsen B. A case for upward continuation as a standard separation filter for potential-field maps. Geophysics. 1987, 52, 1138–1148. 10.1190/1.1442378.

[ref36] Xu H.; Wei J.; Han X.; Zhao F.; Zhou J.; Jiang T.; Shi J.; Xu W.; Song B.; Wang B. Characteristics of gravity and magnetic anomalies and their petroleum geological significance in the Yingen-Ejinaqi basin, Inner Mongolia, China. Journal of Geophysics and Engineering. 2024, 21 (1), 304–329. 10.1093/jge/gxad102.

[ref37] Rosenbach O. A contribution to the “second derivative” from gravity data. Geophysics. 1953, 18, 894–912. 10.1190/1.1437943.

[ref38] Cordell L. Gravimetric expression of graben faulting in Santa Fe Country and the Espanola Basin, New Maxico. New Mexico Geological Society Guidebook, 30th Field Conference. 1979, 59–64.

[ref39] Elkins T. The second derivative method of gravity interpretation. Geophysics. 1951, 16, 29–50. 10.1190/1.1437648.

[ref40] Xu H.; Yuan B.; Yang G.; Liu J.; Zhang C. The application of the small sub-domain filtering combined with the total horizontal derivative to gravity data processing: a case study of fault structure identification in EQ block of Yin-E Basin,Inner Mongolia. Geol. Bull. China 2014, 33 (11), 1853–1860.

[ref41] Xu H.; Li Y.; Yuan B.; Jiang T.; Wei J.; Zhang C. The processing effect comparison and optimization of small subdomain filtering method in potential field data. Geol. Bull. China 2018, 37 (1), 153–164.

[ref42] Zeng H. Gravity field and gravity exploration; Geological Publishing House, 2005.

[ref43] Zhang C.; Liu S.; Yuan B.; Zhang G. Fault structure and hydrocarbon prospects of the Palawan basin on the southeastern margin of the South China Sea based on gravity, magnetic, and seismic data. Interpretation. 2024, 12 (3), T187–T196. 10.1190/INT-2022-0125.1.

[ref44] Wang Y. Methods for Seismic Data Comprehensive Interpretation; China University of Petroleum Press, 2007.

[ref45] Xu W.; Wei J.; Han W.; Dang B.; Niu Y.; Han X.; Song B.; Xue N. A preliminary study of the structure and reformation of the Permian and Carboniferous strata in Yingen-Ejin basin and its periphery. Geol. Bull. China 2018, 37 (1), 132–143.

[ref46] Lu C.; Duan C.; Kang X.; Li B. Bedrock Hydrocarbon-Bearing Characteristics and Pool-Forming Conditions of Mamuwusu Sag in Badanin Jaran Basin. Exp. Petrol. Geol. 1999, 21 (3), 251–255. 10.11781/sysydz199903251.

[ref47] Lu J.; Wei J.; Jiang T.; Xu H.; Wang B. The physical and chemical characteristics of crude oil and oil-source of Juyanhai depression in Yingen-Ejina Basin. Geol. Bull. China 2020, 39 (10), 1589–1599.

[ref48] Liang S. Achievements and Potential of Petroleum Exploration in Tuha Oil and Gas Province. Xinjiang Petrol. Geol. 2020, 41 (6), 631–641. 10.7657/XJPG20200601.

[ref49] Wei J.; Jiang T.; Wang B.; Zhang Y.; Su Q. The biomarker characteristics and its significance of the Carboniferous- Permian source rocks in Jigeda sag of Juyanhai Depression in Yin’er Basin. NW Geol. 2020, 53 (3), 273–283.

[ref50] Bai Y.; Bo H.; Yang W.; Li C.; Yu Y.; Qin Z. The microfossils and depositional environment from Bayingebi Formation of Lower Cretaceous in the Ejina area of Yingen-Ejin Basin. Geol. Bull. China 2023, 42 (10), 1666–1673.

[ref51] Chen Q.; Yang Z.; Guan Y.; Wei P. Depression structure-style types and petroleum accumulation, rift basin group of Yingen-Ejina. Nat. Gas Geosci. 2005, 16 (5), 559–563.

[ref52] Chen Q.; Zhou H.; Wei P.; Yang Z.; Bai L. Superposed basin types and exploration realms in Mesozoic Yingen-Ejina Basin in the west of NeiMongol. NW Geol. 2006, 39 (1), 89–97.

[ref53] Wu X.; He D.; Chen X.; Zheng M.; Li Y. Geological characteristics and resource potential of the Yingen-Ejin Banner Basin. Chin. J. Geol. 2020, 55 (2), 404–419.

